# Anesthesia interventions that alter perioperative mortality: a scoping review

**DOI:** 10.1186/s13643-018-0863-x

**Published:** 2018-11-30

**Authors:** Sylvain Boet, Cole Etherington, David Nicola, Andrew Beck, Susan Bragg, Ian D. Carrigan, Sarah Larrigan, Cassandra T. Mendonca, Isaac Miao, Tatyana Postonogova, Benjamin Walker, José De Wit, Karim Mohamed, Nadia Balaa, Manoj Mathew Lalu, Daniel I McIsaac, David Moher, Adrienne Stevens, Donald Miller, On behalf of the Perioperative Anesthesia Clinical Trials Group (PACT)

**Affiliations:** 1 Department of Anesthesiology and Pain Medicine, The Ottawa Hospital, University of Ottawa, General Campus, 501 Smyth Rd, Critical Care Wing 1401, Ottawa, Ontario K1H 8L6 Canada; 2 Clinical Epidemiology Program, Ottawa Hospital Research Institute, Ottawa, Canada; 3 Family Medicine, McGill University, Montreal, Canada; 4 Knowledge Synthesis Group, Clinical Epidemiology Program, Ottawa Hospital Research Institute, Ottawa, Canada; 5 Department of Anesthesia, University of Toronto, Toronto, Canada; 6 Faculty of Medicine, University of Ottawa, Ottawa, Canada; 7 University of Ottawa, Ottawa, Canada; 8 Department of Anesthesiology, McGill University, Montreal, Quebec Canada; 9 Department of Anesthesia, University of Utah, Salt Lake City, UT 84132 USA; 10 Department of Family Medicine, Montfort Hospital, University of Ottawa, Ottawa, Canada; 11 Department of Anesthesiology and Pain Medicine, The Ottawa Hospital, Ottawa, Canada; 12 Clinical Epidemiology and Regenerative Medicine Programs, Ottawa Hospital Research Institute, Ottawa, Canada; 13 School of Epidemiology and Public Health, University of Ottawa, Ottawa, Ontario K1G 5Z3 Canada

**Keywords:** Anesthesiology, Mortality, Review

## Abstract

**Background:**

With over 230 million surgical procedures performed annually worldwide, better application of evidence in anesthesia and perioperative medicine may reduce widespread variation in clinical practice and improve patient care. However, a comprehensive summary of the complete available evidence has yet to be conducted. This scoping review aims to map the existing literature investigating perioperative anesthesia interventions and their potential impact on patient mortality, to inform future knowledge translation and ultimately improve perioperative clinical practice.

**Methods:**

Searches were conducted in MEDLINE, EMBASE, CINAHL, and the Cochrane Library databases from inception to March 2015. Study inclusion criteria were adult patients, surgical procedures requiring anesthesia, perioperative intervention conducted/organized by a professional with training in anesthesia, randomized controlled trials (RCTs), and patient mortality as an outcome. Studies were screened for inclusion, and data was extracted in duplicate by pairs of independent reviewers. Data were extracted, tabulated, and reported thematically.

**Results:**

Among the 10,505 publications identified, 369 RCTs (*n* = 147,326 patients) met the eligibility criteria. While 15 intervention themes were identified, only 7 themes (39 studies) had a significant impact on mortality: pharmacotherapy (*n* = 23), nutritional (*n* = 3), transfusion (*n* = 4), ventilation (*n* = 5), glucose control (*n* = 1), medical device (*n* = 2), and dialysis (*n* = 1).

**Conclusions:**

By mapping intervention themes, this scoping review has identified areas requiring further systematic investigation given their potential value for reducing patient mortality as well as areas where continued investment may not be cost-effective given limited evidence for improving survival. This is a key starting point for future knowledge translation to optimize anesthesia practice.

**Electronic supplementary material:**

The online version of this article (10.1186/s13643-018-0863-x) contains supplementary material, which is available to authorized users.

## Background

### Rationale

With more than 230 million major surgical procedures performed annually worldwide [1], better application of evidence in anesthesia and perioperative medicine has the potential to significantly improve patient safety, care, and satisfaction [2, 3]. Currently, as with many other specialities, anesthesiologists have large variations in practice and patient outcomes [4–7], with many examples of failure to follow best practices [8, 9]. For instance, the maintenance of perioperative normothermia has been shown to decrease the rate of surgical site infection by threefold [10–15]. However, while effective tools for maintaining normothermia exist, perioperative hypothermia continues to affect up to 20% of patients [15].

Clearly, the mere existence of best clinical evidence does not mean that it will be adopted into practice or improve patient outcomes [16]. Knowledge translation (KT) [17, 18] focuses on the effective implementation of best clinical practices, moving evidence to practice [19]. In anesthesiology, there have been few previous attempts to summarize comprehensively the broader peer-reviewed literature and those articles which have been published face methodological limitations [20, 21]. A comprehensive summary of the complete available evidence related to mortality has yet to be conducted.

Scoping reviews have been specifically described as a process of mapping the existing evidence, by providing a comprehensive and thorough review of the available literature [22]. They are particularly useful for complex fields, such as anesthesiology and perioperative medicine [23]. Unlike systematic reviews, scoping reviews summarize a range of evidence in order to convey the breadth and depth of evidence in a certain field [23]. In addition, scoping studies differ from systematic reviews because they address broad research questions and do not typically quantify the effect of interventions [24, 25]. Instead, a scoping review examines the extent, range, nature, and characteristics of the primary research and summarizes the evidence [22]. Scoping reviews are often preliminary to full systematic reviews when the field of research is broad and complex, such as anesthesiology and perioperative medicine, making the feasibility of a systematic review a concern because the potentially relevant literature is thought to be especially vast and diverse.

## Methods

This scoping review is reported according to the PRISMA Extension for Scoping Reviews (PRISMA-ScR) Checklist [26]. We also followed the current framework developed by Arksey and O’Malley [22] and Levac et al. [27] in conducting this review. In order to achieve the purposes of the scoping review, we employed a five-stage framework: (1) identify the research question, (2) identify relevant published studies, (3) refine the study selection criteria, (4) collect the relevant data from each published article, and (5) collate, summarize, report, and interpret the results.

### Objectives

Based on the existing gaps in the literature, we asked the following research question: What types of anesthesia-related interventions impact patients’ mortality?

### Eligibility criteria

Based on the existing gaps in the literature, we asked the following research question: What types of anesthesia-related interventions impact patients’ mortality? We selected all articles published in journals that involved adult patients (≥ 16 years old) undergoing surgery and that evaluated an anesthesia-related intervention. Anesthesia-related interventions were defined as interventions provided in the perioperative period that either were, or could have been, performed, organized, or initiated by a healthcare professional with specific training in anesthesia. For example, the following interventions were included: perioperative administration of antibiotherapy [28], intraoperative remote ischemic preconditioning [29], and postoperative ventilation support [30]. Furthermore, studies involving surgical procedures involving local anesthesia only were excluded, as well as studies reporting perioperative interventions that are exclusively interested in comparing different surgical techniques (e.g., laparoscopic versus open surgery). The perioperative period referred to the time window from the initial preoperative anesthesia assessment before the surgery to the final care provided or organized by anesthesia providers following surgery. Therefore, the perioperative period was separated into three distinct phases: preoperative, intraoperative, and postoperative. We focused on the studies assessing mortality as an outcome and included randomized controlled trials (RCTs). The comparator group of the RCT was defined as either no treatment or usual standard of care. We did not impose a minimum sample size for included studies.

### Information sources and search strategy

The initial search strategy was developed with the active contribution of experts in the methodology of conducting reviews (AS), a practicing anesthesiologist (SB), and a health sciences librarian (LAH).

The electronic databases MEDLINE, EMBASE, CINAHL, and the Cochrane Library were searched. Literature searches were performed without any language restrictions, but we only included articles published in English. The literature search was performed on March 5, 2015, without any year restriction. The MEDLINE search strategy underwent Peer Review of Electronic Search Strategies (PRESS) by a second trained information scientist [31, 32]. Search strategies can be found in Additional file 1. Reference lists of relevant systematic reviews were also searched to identify additional relevant studies. The final list of the included studies was also reviewed by the Perioperative Anesthesia Clinical Trials Group (PACT) for both completeness and relevance.

### Selection of sources of evidence

The identified articles were merged into the web-based systematic review software DistillerSR (Evidence Partners, Ottawa, Canada), and duplicates were removed. The screening tools were developed by the research team and piloted with a subset of articles for refinement and reviewer calibration. Reviewers were trained on how to use DistillerSR (Evidence Partners, Ottawa, Canada) and to critically appraise articles according to the inclusion and exclusion criteria of this scoping review.

Articles were screened for eligibility by title and abstract by two individuals using the liberal accelerated screening approach [33]. This approach involved one reviewer screening citations by title and abstract using the screening tool based on the predetermined criteria. The selected studies classified as either “included” or “unclear” advanced to the subsequent screening stage. However, studies classified as “excluded,” were reviewed by the other reviewer to determine whether the exclusion criteria were properly met.

Following the completion of the title and abstract screening stage, the full texts of all qualified studies were reviewed in duplicate by six pairs of independent reviewers (DN, AB, SB, IC, CM, IM, TP, BW, SL, KM, JDW, NB) for compliance with eligibility criteria. Disagreements were resolved by consensus or referred to a third member of the research team for resolution. The list of included articles was reviewed by the investigators to determine if any additional articles should be included [34].

The accuracy of the included and excluded studies was verified using the artificial intelligence feature of DistillerSR (Evidence Partners, Ottawa, Canada).

### Data charting process

A data extraction form was created and piloted by the research team. Six pairs of reviewers (DN, AB, SB, IC, CM, IM, TP, BW, SL, KM, JDW, NB) independently extracted the study characteristics and the mortality outcome from the included studies. We collected information on publication details (e.g., first author’s name, year of publication, study location) and information about the study details (e.g., study design; sample size; gender; age; ASA score; intervention details, such as duration and type; setting; perioperative phase; anesthesia type; comparator; and mortality outcome details, such as timing). The significance of the intervention on mortality is reported according to how it was defined by the study authors.

Where data was inadequately reported within the full-text article, we contacted the original authors for clarification and further details. Quality assessments of included studies were not reported because they are typically not completed during the scoping reviews [22].

### Data items

This scoping review allowed for the development of anesthesia-related intervention themes that outline what evidence levels currently exist as well as the potential gaps in anesthesia research that may be further explored. Interventions were classified according to these themes and defined in Additional file 2. A list of themes was determined a priori, and reviewers could add new themes when a study did not fit any a priori theme.

### Synthesis of results

The results of this scoping review were synthesized using both a numerical summary outlining the relevant characteristics of the included studies and a narrative synthesis interpreting the results (Additional files 3 and 4).

## Results

### Study selection

**Fig. 1:**
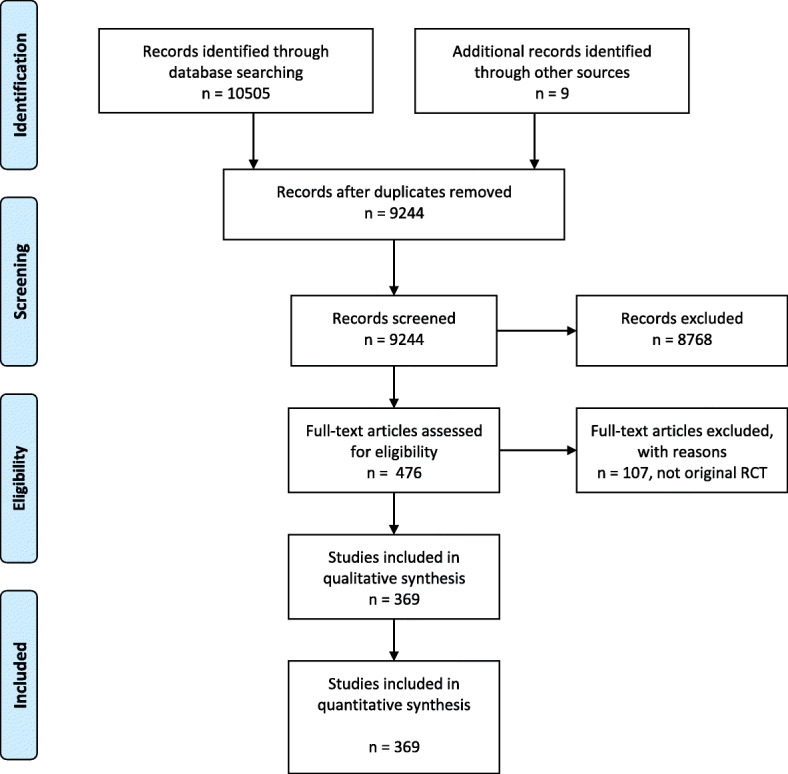
PRISMA diagram

The literature search strategy yielded a total of 10,505 references, of which 1270 were duplicates. Nine additional studies were identified: eight from the reference list of a relevant systematic review and one identified by experts. After screening, 8768 references were excluded. A total of 369 references met the inclusion criteria (Fig. 1). The AI feature on DistillerSR (Evidence Partners, Ottawa, Canada) confirmed that all inclusions and exclusions were correct (i.e., the AI system did not identify any articles erroneously included or excluded by the human screeners).

### Study characteristics

**Fig. 2:**
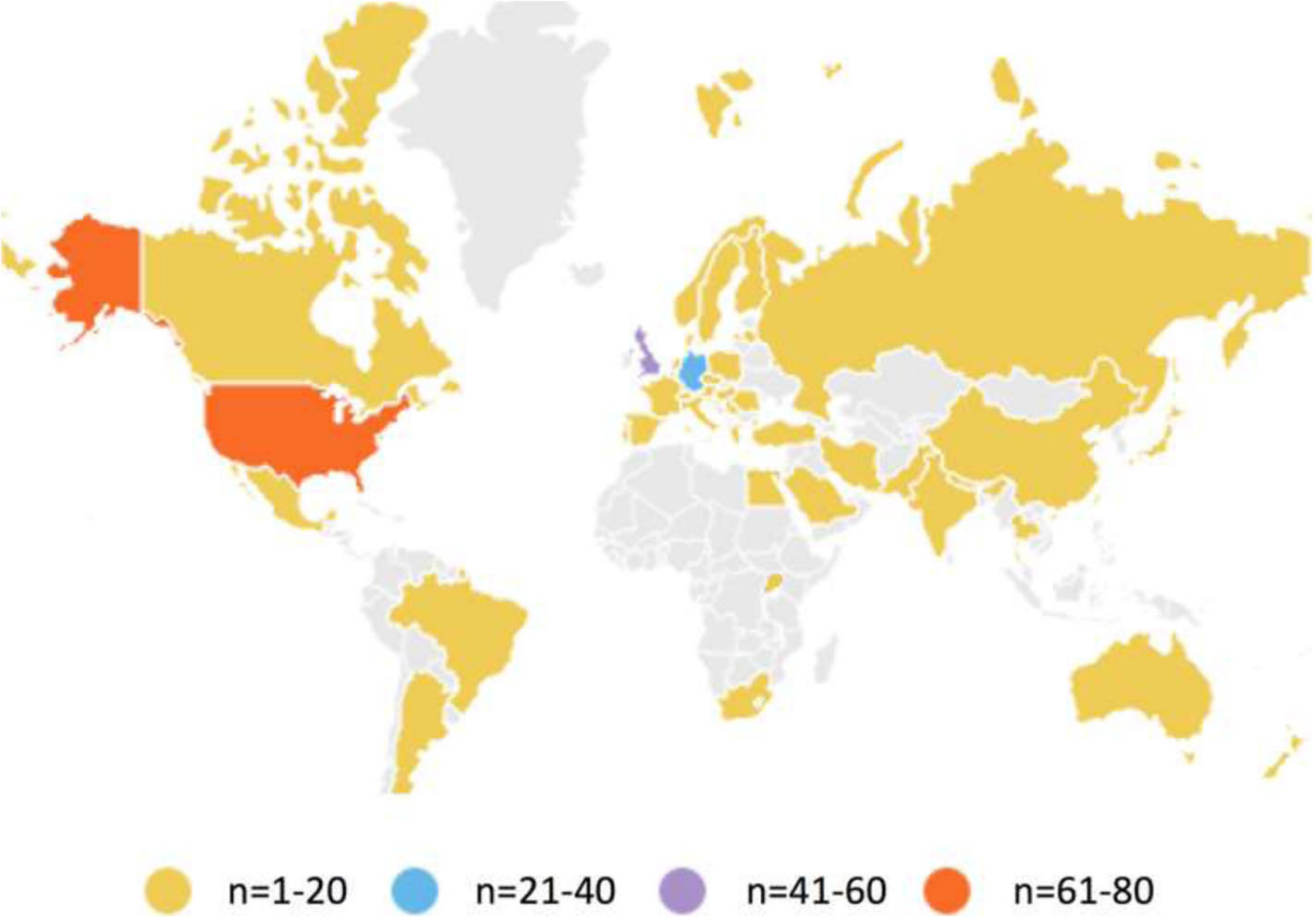
Country of data collection for anesthesia-related interventions

Details of the included study characteristics, participants, type of surgery, study design, and interventions are provided in Additional file 1. Of the 369 studies, 331 indicated data collection occurred in a single country. These locations are displayed in Fig. 2. One study did not report country of data collection. The remaining 37 studies indicated data collection was performed in more than one country. The largest number of trials was conducted in the USA (*n* = 69). Multicenter trials were conducted in 102 studies, with the remainder involving a single center. The 369 trials included a total of 147,326 participants (median 123, IQR 60–272).

#### Anesthesia-related intervention theme, type of surgery and perioperative phase

The most common anesthesia-related intervention theme found was pharmacotherapy (201 included studies [54%]; 104,413 patients [71%]) followed by nutritional interventions (28 studies [8%]; 5191 patients [4%]) (Table 1). Most studies (*n* = 180 studies [49%]; *n* = 95,119 patients [65%]) involved interventions spanning more than one perioperative phase (i.e., various combinations of preoperative, intraoperative, and postoperative phases) (Table 2). The most common type of surgery reported was cardiac surgery (*n* = 151 studies [41%]; *n* = 63,738 patients [43%]), followed by general surgery (*n* = 66 studies [18%]; *n* = 13,458 patients [9%]) and vascular surgery (*n* = 31 studies [8%]; *n* = 6404 patients [4%]).

**Table 1:** Anesthesia-related intervention themes by number of studies and patients

Intervention category	Number of studies	Number of patients
Anesthetic technique	13	2445
Dialysis	1	44
Glucose control	6	2139
IV fluids	13	1869
Medical device	21	5233
Monitoring	4	3341
Nutritional	28	5191
Pharmacotherapy	207	104,413
Physiotherapy	2	283
Preoperative procedure	1	510
Protocol/guidelines implementation	22	2705
Temperature management	4	2444
Testing	2	1566
Transfusion	23	8747
Ventilation	18	5469
Combination of interventions	4	927
Total	369	147,326

**Table 2:** Perioperative phase of anesthesia-related interventions according to the number of studies and patients

Perioperative phase	Number of studies	Number of patients
Preoperative	25	6139
Intraoperative	101	24,824
Postoperative	62	20,964
Multiphase (i.e., intervention spanned across 2 or 3 phases)	180	95,119
Not reported	1	280
Total	369	147,326

### Mortality outcome of anesthesia-related interventions

The vast majority of included studies showed no statistically significant effect on mortality (*n* = 330 [89%] studies; *n* = 117,905 [80%] patients).

Only 39 studies (11%) representing 29,421 patients (20%) reported a significant difference in mortality, with either a decrease (*n* = 30 [77%] “significant” studies; *n* = 10,660 [36%] patients), an increase (*n* = 8 [21%] studies; *n* = 18,459 [63%] patients), or both depending on the time at which mortality was measured (*n* = 1 [3%] study; *n* = 302 [1%] patients). The following intervention themes were represented: pharmacotherapy (*n* = 23 [59%] studies; *n* = 23,322 [79%] patients), nutritional (*n* = 3 [8%] studies; *n* = 797 [3%] patients), transfusion (*n* = 4 [10%] studies; *n* = 1558 [5%] patients), ventilation (*n* = 5 [13%] studies; *n* = 1602 [5%] patients), medical device (*n* = 2 [5%] studies; *n* = 550 [2%] patients), dialysis-related (*n* = 1 [3%] study; *n* = 44 [0.1%] patients), and glucose control (*n* = 1 [3%] study; *n* = 1548 [5%] patients).

### Interventions that impact survival

**Fig. 3:**
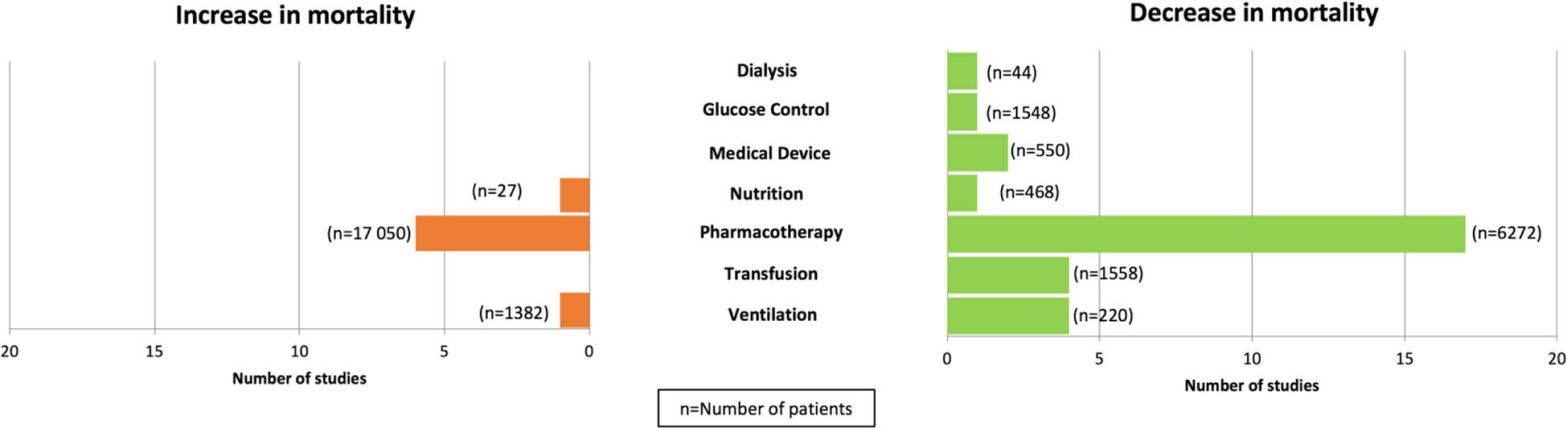
Impact of anesthesia-related interventions on mortality

Only seven themes were represented among the anesthesia interventions that were associated with altered mortality rates (Fig. 3). For each intervention theme, we discuss only those studies that reported a statistically significant effect on patient mortality.

#### Pharmacotherapy interventions

As shown in Table 3, a total of 23 publications (23,322 patients) were identified for anesthesia-related pharmacotherapy interventions which significantly impacted mortality. Of these studies, 6 (26%) reported an increase in mortality (*n* = 17,050 [73%] patients) while 17 (74%) reported a decrease in mortality (*n* = 6272 [27%] patients). Most studies that reported a significant impact on mortality (*n* = 15 [65%]; *n* = 13,368 [57%] patients) reported mortality as a primary outcome. The most frequent type of surgery involved was cardiac (*n* = 10 [43%]; *n* = 12,045 [52%] patients). Pharmacotherapy interventions involved a wide variety of pharmacotherapy and dosing regimens, with the interventions most commonly occurring across more than one surgical phase (*n* = 18 [78%]; *n* = 13,302 [57%] patients).

**Table 3:** Summary of mortality outcome for pharmacotherapy interventions for a significant difference in mortality

First author, year	Type of surgery, no. of participants	Intervention/comparison details	Perioperative phase, duration of intervention	Impact on mortality* (outcome definition, timing)
Aronson, 2008	Cardiac, 1506	IV clevidipine at an initial rate of 0.4 mcg/kg/min, titrating to antihypertensive effect to a max dose of 8 mcg/kg/min. Three comparator groups of common (usual care) perioperative antihypertensives: nitroglycerin, sodium nitroprusside, and nicardipine.	Preoperative, intraoperative, postoperative Once	Decreased mortality (death at 30 days, primary outcome)
Boyd, 1993	Major surgery, 107	Dopexamine infusion to achieve oxygen delivery (DO_2_I) of greater than 600 mL/min/m^2^, perioperatively, in high-risk patients. Usual care	Preoperative, intraoperative, postoperative 24 h	Decreased mortality (in-hospital mortality, primary outcome)
Comerota, 1993	Vascular, 134	One of three doses of urokinase (125,000, 250,000, or 500,000) infused into the distal circulation before lower extremity bypass for chronic limb ischemia No treatment	Intraoperative Once	Increased mortality (death at NR, secondary outcome)
Devereaux, 2008	Non-cardiac, 8351	Extended-release metoprolol 2–4 h before surgery and continued for 30 days Placebo	Preoperative, postoperative Once	Increased mortality (cardiovascular death, NR, primary outcome)
Donato, 2007	Vascular, 192	Iloprost (intra-arterial, intraoperative bolus) of 3000 ng, plus intravenous infusion of 0.5–2.0 ng/kg/min. No treatment	Intraoperative, postoperative Every day for a time period	Decreased mortality (mortality at 90 days, primary outcome)
Donato, 2006	Vascular, 300	Starting from the first day after surgery, a daily 6-h intravenous infusion of iloprost (or placebo) at doses recommended for chronic critical limb ischemia was performed for 4 to 7 days (7 days recommended). No treatment	Intraoperative, postoperative Every day for a time period	Decreased mortality (mortality at 90 days, primary outcome)
Fergusson, 2008	Cardiac, 2331	Aprotinin: test dose of 40,000 KIU administered during a 10-min period after insertion of central venous line and induction of anesthesia. If no anaphylactic reaction remained for loading dose (1.96 million KIU) given followed by maintenance infusion of 500,000 KIU/h and maintained during surgery. Aminocaproic acid or tranexamic acid	Intraoperative During most of the intraoperative period	Increased mortality (death from all causes at 30 days, secondary outcome)
Giakoumidakis, 2013	Cardiac, 200	Group 1 received aspirin preoperatively while in group 2, aspirin was stopped at least 7 days before CABG. No treatment	Preoperative Once	Decreased mortality (in-hospital mortality, primary outcome)
Hase, 2013	Cardiac, 350	Bolus of sodium bicarbonate (0.5 mmol/kg in 250 mL over 1 h) at induction followed by an infusion over the next 23 h (0.2 mmol/kg/h in 1000 mL).	Intraoperative, postoperative 24 h	Increased mortality (death in-hospital, secondary outcome) ND (death at 90 days, secondary outcome)
Herr, 2000	NR, 113	Propofol or propofol plus EDTA	Intraoperative Once	Increased mortality (7-day mortality, primary outcome)
Iliuta, 2009	Cardiac, 1352	Group A: patients with betaxolol postoperative 20 mg once daily Group B: patients with metoprolol postoperative 200 mg in two equal doses daily	Preoperative, intraoperative postoperative, after discharge from hospital Every day for a time period	Decreased mortality (30-day mortality, primary outcome)
Illiuta, 2003	Cardiac, 400	Patients received nadroparin 85 U/kg SC q12h. Usual care: patients received unfractionated heparin IV to maintain APTT at 2.5 the normal value.	Postoperative Every day for a time period	Decreased mortality (30-day mortality, primary outcome)
Kirdemir, 2008	Cardiac, 200	Continuous insulin infusion titrated per protocol in the perioperative period (Portland protocol) to maintain blood glucose between 100 and 150 mg/dL. Subcutaneous insulin was injected every 4 h in a directed attempt to maintain blood glucose levels below 200 mg/dL.	Intraoperative, postoperative Immediately preoperatively until postop day 3	Decreased mortality (in-hospital mortality, secondary outcome)
Krestchmer, 1989	Vascular, 252	ASA (1–1.5 g daily) No treatment	Preoperative, postoperative, after discharge from hospital Every day for a time period	Decreased mortality (probability of survival at 6 years, primary outcome)
Levin, 2012	Cardiac, 93	Preoperative loading dose of levosimendan (10 μg/kg over 60 min) followed by a continuous 23 h infusion of 0.1 μg/kg/min No treatment	Preoperative Every day for a time period	Decreased mortality (30-day mortality, primary outcome)
Levin, 2008	Cardiac, 252	Preoperative loading dose of levosimendan (10 μg/kg over 60 min) followed by a continuous 23 h infusion of 0.1 μg/kg/min No treatment	Preoperative, intraoperative Every day for a time period	Decreased mortality (30-day mortality, primary outcome)
Mentzer, 2008	Cardiac, 5761	Intravenous cariporide (180 mg in a 1-h preoperative loading dose, then 40 mg/h over 24 h and 20 mg/h over the subsequent 24 h). No treatment	Preoperative	Increased mortality (all-cause mortality at day 5, secondary outcome) Increased mortality (all-cause mortality at day 30, secondary outcome) ND (all-cause mortality at 6 months, secondary outcome)
Norman, 2009	Thoracic, 16	Aprotinin (IV bonus of 2 million KIU followed by a 0.5 million KIU per but infusion). No treatment	Intraoperative Once	Decreased mortality (survival at NR, secondary outcome)
Poldermans, 1999*	Vascular, 112	Beta-blockade with bisoprolol Usual care with no perioperative blockade	Preoperative, intraoperative postoperative Until surgery	Decreased mortality (perioperative death, primary outcome)
Reyad, 2013	General, 60	Dobutamine at either 3 mcg/kg/min or 5 mcg/kg/min. No treatment	Intraoperative During most of the intraoperative phase	Decreased mortality (death in-hospital, secondary outcome)
Turpie, 2007	General, 467	Injections of fondaparinux 2.5 mg (fondaparinux sodium, Arixtra, GlaxoSmithKline, Research Triangle Park, NC, USA). No treatment	Postoperative Every day for a time period: daily for 5–9 days	Decreased mortality (death at 30 days, secondary outcome)
Wallace, 2004	General, 190	0.2 mg oral tablet of clonidine (Catapres; Boehringer Ingelheim, Ridgefield, CT), a 7.0-cm^2^ transdermal patch of clonidine (Catapres-TTS-2; Boehringer Ingelheim), providing continuous systemic delivery of 0.2 mg/day, and an oral loading dose of clonidine, 0.2-mg tablet (Catapres). No treatment	Preoperative, intraoperative, postoperative Every day for a time period: 4 days	Decreased mortality (30-day mortality, NR) Decreased mortality (2-year mortality, NR)
Wilson, 1999	General, 138	1 L of Hartmann’s solution during line insertion. Human albumin solution 4.5% was then infused until a pulmonary artery occlusion pressure of 12 mmHg was achieved. If hemoglobin concentration was < 110 g/L, red blood cells were transfused instead of the albumin solution. If oxygen saturation was < 94%, supplemental oxygen was provided. Inotrope was commenced at a rate (mL/h) calculated from a chart according to the patient’s weight and equated to 0.025 ìg/kg/min for adrenaline. The infusion was increased by single multiples of the initial rate until the target oxygen delivery of > 600 ml/min/m^2^ was achieved or the onset of side effects was noted (increase in heart rate > 30% above baseline or development of chest pain or a new dysrhythmia). All patients were started on the study inotrope even if the target oxygen delivery had been achieved after the fluid phase. Usual care	Preoperative, intraoperative, postoperative Minimum of 4 h before surgery, continued for at least 12 h afterwards.	Decreased mortality (in-hospital mortality, primary outcome)

Anesthesia-related intervention refers to the interventions provided in the perioperative period that was or could be performed, organized, or initiated by a healthcare professional with specific training in anesthesia

*ND* no significant change, *NR* not reported

*This study was part of an investigation of academic integrity. The investigating committee was unable to confirm or deny any doubts surrounding the conduct of the study, and it thus not retracted from the journal where it was published. We therefore did not exclude the study from our scoping review

#### Nutritional interventions

Table 4 reports the three nutritional interventions (*n* = 797 patients), identified as having a significant impact on mortality. Of these studies, one (33%) reported an increase in mortality (*n* = 27 [3%] patients) [35], one (33%) reported a decrease in mortality (*n* = 468 [59%] patients) [36], and one (33%) reported an increase in mortality in-hospital, but a decrease in deaths occurring at 4 months (*n* = 302 [38%] patients) [37]. Each study implemented a distinct nutritional intervention, and each involved a different field of surgery (thoracic, orthopedic, cardiac, colorectal). Mortality was reported as a primary outcome in two of the studies, with both finding an increase in mortality. Of the three different nutritional interventions, two were given pre- and postoperatively and one was given postoperatively only.

**Table 4:** Summary of mortality outcome for nutritional interventions for significant difference in mortality

First author, year	Type of surgery, no. of participants	Intervention/comparison details	Perioperative phase, duration of intervention	Impact on mortality* (outcome definition, timing)
Cooper, 2006	Thoracic-oncological, 27	Total parenteral nutrition Usual care: maintaining patients NPO until postop day 4, then initiating an oral diet	Preoperative, postoperative Every day for a time period	Increased mortality (90-day mortality, primary outcome) ND (1-year mortality, primary outcome)
Duncan, 2005	Orthopedic, 302	Feeding support by dietetic assistants Usual care: traditional nurse- and dietitian-led nutrition and feeding postop	Postoperative Every day for a time period until discharge	Increased mortality (death in trauma unit, primary outcome) Decreased mortality (death at 4 months, secondary outcome) ND (death in-hospital, secondary outcome)
Wu, 2006	Colorectal, 468	7 days preop and 7 days postop either parenteral or enteral nutrition Usual care: usual hospital diet preop and then hypocaloric parenteral solution postop	Preoperative, postoperative Every day for a time period	Decreased mortality (in-hospital mortality, NR)

*ND* no significant change, *NR* not reported

#### Transfusion interventions

As shown in Table 5, four transfusion interventions demonstrated a significant decrease in mortality (*n* = 1558 patients). Two studies involved transfusion of leuko-depleted red blood cells during cardiac surgery [38, 39]. Each study assessed mortality as a secondary outcome, either in-hospital or until 60 days following surgery. Another study [40] involved orthopedic surgery and the perioperative transfusion of hemoglobin at a threshold of 10.0 g/dL. Mortality was assessed as a secondary outcome at 30 days. The final study [41] involved pre- and postoperative administration of subcutaneous erythropoietin (r-HuEPO at 300 IU/kg) plus IV iron (100 mg). Survival at 1 year was assessed, but it was not reported whether this was a primary or secondary outcome.

**Table 5:** Summary of mortality outcome for transfusion interventions for a significant difference in mortality

First author, year	Type of surgery, no. of participants	Intervention/comparison details	Perioperative phase, duration of intervention	Impact on mortality* (outcome definition, timing)
Bilgin, 2004	Cardiac, 474	Leuko-depleted red blood cell transfusion. Platelet concentrates were prepared from pooled buffy coats and were all leukocyte-depleted by filtration (< 1 × 10^6^ leukocytes per product) before storage. Standard buffy coat-depleted packaged cells.	Intraoperative During most of the intraoperative phase	Decreased mortality (in-hospital, secondary outcome)
Foss, 2009	Orthopedic, 107	A hemoglobin threshold of 10.0 g/dL (liberal) versus 8.0 g/dL (restrictive) in the entire perioperative period Receive transfusion at a hemoglobin threshold of 8.0 g/dL (restrictive) in the entire perioperative period.	Preoperative, Intraoperative, postoperative During most of the intraoperative phase	Decreased mortality (30-day mortality, secondary outcome)
Kosmadakis, 2003	Colorectal, 63	The intervention involved administration of subcutaneous erythropoietin (r-HuEPO at 300 IU/kg) plus IV iron (100 mg) for 7 days preop, and 7 days postop surgery for gastrointestinal malignancies. Received placebo medication subcutaneously and 100 mg iron intravenously each day.	Preoperative, postoperative Every day for a time period: “erythropoietin or placebo applications were given for 14 days perioperatively, starting 7 days before the operation.”	Decreased mortality (1-year survival, NR)
Van de Watering, 1998	Cardiac, 914	One of the following three trial arms: “(1) the PC trial arm, in which when transfusion was indicated, standard packed cells (PC) without buffy coat were transfused; (2) the prestorage filtration FF trial arm, in which when transfusion was indicated, freshly filtered (i.e., < 24 h after donation) units were transfused; and (3) the poststorage filtration SF trial arm, ill which when transfusion was indicated, 6- to 20-day stored packed cells without buffy coat were filtered shortly before transfusion.”	Intraoperative During most of the intraoperative phase	Decreased mortality (in-hospital, NR)

Anesthesia-related intervention refers to interventions provided in the perioperative period that was or could be performed, organized, or initiated by a healthcare professional with specific training in anesthesia

*ND* no significant change, *NR* not reported

#### Ventilation interventions

Five ventilation interventions (*n* = 1602 patients) had a statistically significant impact on mortality. Two were administered intraoperatively and postoperatively [42, 43], and three were administered only postoperatively [30, 44, 45] (Table 6). Non-invasive ventilation decreased in-hospital mortality in three studies (*n* = 183 [11%] patients) [30, 44, 45]. It was also found by one study to decrease mortality at 120 days [30]. The intervention which decreased mortality at 60 days (*n* = 37 [2%] patients) involved intentionally increasing oxygen delivery to > 600 ml/min/m^2^ [42]. The intervention which increased mortality at 2 years (*n* = 1382 [86%] patients) involved giving patients an FiO_2_ of 0.80 after intubation and for 2 h after surgery [43]. All but one study [30] assessed mortality as a primary outcome.

**Table 6:** Summary of mortality outcome for ventilation interventions for a significant difference in mortality

First author, year	Type of surgery, no. of participants	Intervention/comparison details	Perioperative phase, duration of intervention	Impact on mortality* (outcome definition, timing)
Antonelli, 2000	General or Thoracic, 40	Non-invasive ventilation: “…the ventilator was connected with conventional tubing to a clear, full face mask with an inflatable soft cushion seal and a disposable foam spacer to reduce dead space. After the mask was secured, pressure support was increased to obtain an exhaled tidal volume of 8 to 10 mL/kg, a respiratory rate of fewer than 25 per minute, the disappearance of accessory muscle activity (as evaluated by palpating the sternocleidomastoid muscle), and patient comfort. Positive end-expiratory pressure was increased in increments of 2 to 3 cm H2O repeatedly up to 10 cm H2O until the FiO_2_ requirement was 0.6 or less.” Standard treatment with supplemental oxygen administration	Postoperative “During the first 24 h, ventilation was continuously maintained until oxygenation and clinical status improved. Subsequently, each patient was evaluated daily while breathing supplemental oxygen without ventilatory support for 15 min. Non-invasive ventilation was reduced progressively in accordance with the degree of clinical improvement and was discontinued if the patient maintained a respiratory rate lower than 30 per minute and a PaO_2_ greater than 75 mmHg with a FiO_2_ of 0.5 without ventilatory support.”	Decreased mortality (rate of fatal complications, in-hospital, primary outcome)
Auriant, 2001	Thoracic, 48	NPVV: “Ventilation was provided via a cushion bridge nasal mask (Profil lite; Respironics.Inc., Murrysville, PA). NPPV was provided with the BiPAP S/T-D Ventilatory Support System (Bipap Vision; Respironics, Inc.). Pressure support was increased to achieve an exhaled tidal volume of 8 to 10 mL/kg and a respiratory rate of less than 25 breaths per minute. The FiO_2_ was adjusted to obtain a percutaneous oxygen saturation above 90%.” Standard treatment: “All patients received oxygen supplementation to achieve an SaO_2_ above 90%, bronchodilators (aerosolized albuterol), patient-controlled analgesia (PCA) (bolus dose = 1 mg morphine, lockout interval 7 min, maximum hourly dose = 7 mg), and chest physiotherapy.”	Postoperative “The duration of ventilation was standardized according to Wysocki and coworkers.”	Decreased mortality (in-hospital and 120 days, secondary outcomes)
Lobo, 2000	Major oncological or vascular surgery, 37	Increased oxygen levels to > 600 ml/min/m^2^ in patients post-major oncological or vascular surgery. Control group maintained oxygen delivery at 520–600 ml/min/m^2^	Intraoperative, postoperative For the first 24 h of postop ICU admission	ND (28-day mortality, primary outcome) Decreased mortality (60-day mortality, primary outcome)
Meyoff, 2012	General, 1382	After tracheal intubation, patients were given an FiO_2_ of 0.80 or 0.30 according to the randomization Usual care: receive 30% oxygen during and for 2 h after surgery	Intraoperative, postoperative During most of the intraoperative phase	Increased mortality (all-cause mortality at 2 years, primary outcome)
Zhu, 2013	Cardiac, 95	Non-invasive positive pressure ventilation (NPPV). “NPPV therapy was administered using the bilevel positive airways pressure (BiPAP) S/T mode (Resmed, VPAP III, Australia) via a properly fitted face mask (ZS-MZ-, Zhongshan Technique Development Co., Shanghai)… The initial inspiratory pressure (IPAP) was set at 12 cmH_2_O… According to clinical efficacy and patient tolerance, we raised the IPAP and (or) EPAP by 2–3 cmH_2_O every 5 to 10 min…All patients continued to receive NPPV except for coughing, eating, and talking until their condition was improved. Then NPPV was administered intermittently and the IPAP/EPAP was decreased gradually.” “Standard medical care and oxygen therapy as needed.”	Postoperative Until the condition improved.	Decreased mortality (in-hospital mortality, primary)

Anesthesia-related intervention refers to interventions provided in the perioperative period that was or could be performed, organized, or initiated by a healthcare professional with specific training in anesthesia

*ND* no significant change, *NR* not reported

#### Other significant interventions

Four studies investigating three other anesthesia-related interventions that statistically significantly impacted mortality are shown in Table 7 (*n* = 2142 patients). These included use of a device (*n* = 2 [50%], *n* = 550 [26%] patients) [46, 47], dialysis (*n* = 1 [25%], *n* = 44 [2%] patients) [48], and glucose control (*n* = 1 [25%], *n* = 1548 [72%] patients) [49]. Intraoperative devices significantly decreased mortality in-hospital, at 30 days and at 1 year for patients who underwent cardiac surgery. Mortality was assessed as a secondary outcome in one study [46], while the other did not report whether it was a primary or secondary outcome [47]. The dialysis intervention was implemented pre- and postoperatively, involved cardiac surgery, and showed decreased in-hospital mortality. It was not specified whether mortality was considered a primary or secondary outcome. Finally, the glucose control intervention was implemented postoperatively, did not report the type of surgery, and decreased mortality (primary and secondary outcome).

**Table 7:** Summary of mortality outcome for device, dialysis, and glucose control interventions for a significant difference in mortality

First author, year	Type of surgery	Anesthesia-related intervention theme	Intervention/ comparison details	Perioperative phase, duration of intervention	Impact on mortality* (outcome definition, timing)
Durmaz, 2003	Cardiac, 44	Dialysis	Prophylactic preoperative hemodialysis for patients undergoing CABG surgery with underlying renal failure. Usual care: received postoperative dialysis if there was a 50% increase in serum creatinine from baseline or patient exhibited inadequate urine output less than 400 mL for 24 h despite correction of hemodynamic status and diuretic therapy.	Preoperative, postoperative Every day for a time period	Decreased mortality (in-hospital mortality, NR)
Thielman, 2013	Cardiac, 329	Medical device	Remote ischemic preconditioning took place after induction of anesthesia and before skin incision. Three cycles of 5 min ischemia, achieved by inflation of a blood pressure cuff to 200 mmHg, followed by 5 min reperfusion while the cuff was deflated were applied to the upper left arm. No treatment	Intraoperative Once	Decreased mortality (all-cause 30-day mortality, secondary outcome)
Qiu, 2009	Cardiac, 221	Medical Device	“The IABP catheter used was 8 F 34 ml balloon Percor STAT-DL Catheter (Datascope Corp, Fairfield, NJ) connected to a Datascope portable computerized console (Datascope), placed using percutaneous insertion technique via the femoral artery.” “Preoperative insertion was normally performed in the anesthesia preparation room in the operating room (OR) prior to induction of anesthesia.”	Intraoperative During most of the intraoperative period	Decreased mortality (in-hospital, NR)
van den Berghe, 2001	NR, 1548	Glucose control	Intensive insulin therapy (target blood glucose of 80–110 mg/dL) in mechanically ventilated ICU patients Usual care: a continuous infusion of insulin (50 IU in 50 mL 0.9% NaCl) was started only if the blood glucose level exceeded 215 mg/dL, with the infusion adjusted to maintain the level between 180 and 200 mg/dL.	Postoperative In the intervention group, the intensive treatment approach was followed until the patient was discharged from the intensive care unit, at which point the conventional approach was adopted.	Decreased mortality (death during intensive care, primary outcome) Decreased mortality (in-hospital mortality, secondary outcome)

Anesthesia-related intervention refers to interventions provided in the perioperative period that was or could be performed, organized, or initiated by a healthcare professional with specific training in anesthesia

*ND* no significant change, *NR* not reported

## Discussion

This scoping review presents the current evidence relevant to anesthesiologists and policy-makers, highlighting anesthesia-related evidence-based interventions that impact the mortality of adult surgical patients (*n* = 39 RCTs; 29,421 patients). By identifying the nature and distribution of studies as well as the potential value of various anesthesia-related interventions for reducing mortality, this review can be used to identify future directions for perioperative research to translate evidence into practice and improve standardization of care.

Among the 15 themes of anesthesia-related interventions identified, only 7 themes demonstrated a significant impact on mortality: pharmacotherapy, nutrition, transfusion, glucose control, device, dialysis, and ventilation. Each of these themes (with the exception of dialysis) was also identified by Landoni et al. in a recently updated consensus on randomized evidence for the reduction of perioperative mortality [50]. The consensus process followed the first systematic review of RCTs on non-surgical interventions with mortality differences in the operative period [21]. It is therefore a strength of our study to confirm the findings of Landoni et al. [21, 50] and to build upon this important foundation in perioperative research. We also included studies with non-significant findings in our review, which are important for practitioners to consider.

Within the identified anesthesia-related intervention themes, 39 studies (29,421 patients) demonstrated an impact on mortality, either positive or negative. Of the 30 studies where the implemented intervention decreased mortality, 17 involved pharmacotherapies. Thus, pharmacotherapy interventions may have a great potential to reduce mortality among surgical patients. It is interesting to note that the importance of pharmacotherapy on mortality was also found by Landoni et al. [50]. Although they used a different methodology and categorized interventions into different themes, Landoni et al. also found over half of the themes (7/13) related closely to pharmacotherapy. Thus, future research may investigate the generalizability of the effect of specific types of pharmacotherapy and dosages to perioperative survival in a broader variety of surgical populations.

The institutional context where these interventions are applied may also require more systematic reporting and further investigations. For example, McIsaac et al. recently demonstrated that the “hip fracture surgery patients at hospitals that use more than 20 to 25% neuraxial anesthesia have improved survival independent of patient-level anesthesia type and other confounders” [51]. Although broad categorizations of interventions are a useful starting point, one may hypothesize that other traditionally overlooked variables may need to be accounted for to better understand the effect of an intervention. For example, variables such as the type of institution, the implementation process of implementation, or local organization of care may help understanding the part of the effect of an intervention.

In addition to pharmacotherapy, other intervention themes were found promising to reduce mortality: ventilation, transfusion, nutrition, glucose control, dialysis, and medical device. Though these themes had a limited number of studies demonstrating a decrease in mortality, they may be encouraging to investigate further. For example, systematic reviews may explore nutritional or ventilation-related interventions to precisely identify promising practices within these themes that improve morbidity in addition to mortality. This is particularly important given the perceived importance of many of these interventions to practicing clinicians as determined in the Landoni et al. consensus study [50]. Clearly establishing the benefits of these types of interventions is important to facilitate their widespread uptake by clinicians if supported by the available evidence.

Though many promising types of interventions were identified, it is noteworthy that none of the included RCTs investigated the impact of “non-traditional interventions” such as team training [52] or hypnosedation [53] on perioperative mortality. These types of interventions may require more exploratory work in perioperative medicine and could be used in future knowledge translation initiatives if found to be effective. It is also interesting to note that the vast majority of included studies investigated a single type of intervention. Given the low mortality rate in perioperative medicine, at least in high-income Western countries, it may be useful in the future to combine themes and interventions in bundle or multifaceted interventions to further decrease mortality with adequate power.

An additional target for improvement in perioperative research highlighted by our scoping review is standardization in outcome measurement. Even with mortality as an outcome, we observed variability in the definition used based on the time window, cause of death, or mode of data collection considered. A common, well-accepted definition of outcomes may facilitate interpretation of future studies. To this end, several recent initiatives have been launched to tackle the issue of core outcome selection and definition [54–57]. Mortality has been identified as a core outcome measure within these initiatives, but as this scoping review demonstrates, the perioperative and anesthesia-research community must still determine the most effective way to measure and report it. This could have significant implications for interpretations of intervention effectiveness.

Our scoping review should be interpreted in light of several limitations. Firstly, the majority of published studies were not sufficiently powered to observe a statistical impact on mortality; the sample sizes were small, on average. Perioperative mortality is increasingly rare and is estimated at only 0.1 to 0.2% of healthy elective patients when measuring in-hospital mortality [58] and around a 4% 1-year mortality rate after major non-cardiac surgery [59]. While only 39 included studies found a significant impact of the intervention on mortality, this may be due to the fact that most studies included mortality as a non-primary outcome. The lack of power to assess mortality may have resulted in a type II error (i.e., failure to observe a difference where one exists). However, this scoping review should be considered as an initial step to guide the field of perioperative research. Secondly, for feasibility reasons, we elected to include only RCTs that considered mortality outcome. There are of course many other meaningful outcome measures that are clinically relevant to surgical patients. However, mortality may be appropriate as a starting point.

Thirdly, although we followed rigorous and standardized methods, it is likely that relevant studies were missed. Due to the process of conducting systematic and scoping reviews through a literature search strategy and screening, studies could not be included if neither the title nor the abstract mentioned any term related to mortality. For example, some studies mention somewhere in the results section an absence of deaths during the study, although mortality was not reported in the title or abstract, or included in the methods [60]. Nevertheless, when the title, abstract, and/or keywords of a study do not accurately reflect all outcomes examined, this presents a significant challenge for literature analysis in anesthesia and increases the potential for missing studies. It is also possible that some articles may not have had appropriate perioperative subject headings in MEDLINE or perioperative keywords included in the abstract, which would have resulted in them not being retrieved by our search. Future work in anesthesia should aim to accurately index all RCTs. A search filter developed specifically for anesthesia would also be of value and should consider subject headings for the perioperative period that reference preoperative, intraoperative, and postoperative time frames. To mitigate the current problem of standardized literature searching in anesthesia, we used artificial intelligence and expert review.

Finally, there are limitations to the depth of analysis for a scoping review. Therefore, as per scoping review methods, the intention is to map out a field of evidence rather than to thoroughly analyze each trial. The identification of gaps in the existing literature should be cautiously interpreted since the quality of evidence is not typically assessed during scoping reviews [23]. These last two limitations may lead to misleading conclusions about the nature and extent of the gaps in the present research. However, the information reported in this scoping review provides a broad overview about the nature and distribution of studies involved with perioperative anesthesia interventions. Future steps could include systematic reviews on individual themes to provide more specific insight into these questions with a narrower focus.

Future systematic reviews conducted to formally synthesize specific intervention themes identified by this scoping review should examine clinical, methodological, and statistical heterogeneity and conduct meta-analyses as appropriate.

## Conclusion

This scoping review described intervention themes based on existing anesthesia research. As a result, it has identified areas requiring further systematic investigation given their potential value for reducing patient mortality as well as areas where continued investment may not be cost-effective based on limited or no evidence for enhancing patient outcome. Accordingly, this scoping review provides a starting point for future knowledge translation designed to optimize anesthesia practice.

## Additional files

Additional file 1: Search strategies. (DOCX 14 kb)

Additional file 2: Intervention themes and definitions. (DOCX 15 kb)

Additional file 3: Study and population characteristics. (DOCX 127 kb)

Additional file 4: PRISMA Checklist. (PPT 2341 kb)

## Abbreviations

AI: Artificial Intelligence
KT: Knowledge translation
ND: No significant change
NR: Not reported
PRESS: Peer Review of Electronic Search Strategies
RCT: Randomized controlled trial

## References

[1] Weiser TG, Regenbogen SE, Thompson KD (2008). An estimation of the global volume of surgery: a modelling strategy based on available data. Lancet.

[2] Meakins JL, Giobbie-Hurder A, Jonasson O (2006). Evidence-based surgery. Surg Clin North Am.

[3] Hackbarth AD, Hackbarth AD (2012). Eliminating waste in US health care. JAMA.

[4] Beaupre LA, Jones CA, Saunders LD, Johnston DWC, Buckingham J, Majumdar SR (2005). Best practices for elderly hip fracture patients. J Gen Intern Med.

[5] Mazzocco K, Petitti DB, Fong KT (2009). Surgical team behaviors and patient outcomes. Am J Surg.

[6] Pronovost PJ, Rinke ML, Emery K, Dennison C, Blackledge C, Berenholtz SM (2004). Interventions to reduce mortality among patients treated in intensive care units. J Crit Care.

[7] Shehata N, Wilson K, Mazer CD (2007). The proportion of variation in perioperative transfusion decisions in Canada attributable to the hospital. Can J Anesth Can d’anesthésie.

[8] Kalhan R, Mikkelsen M, Dedhiya P (2006). Underuse of lung protective ventilation: analysis of potential factors to explain physician behavior*. Crit Care Med.

[9] Weller JM, Merry AF (2013). I. Best practice and patient safety in anaesthesia. Br J Anaesth.

[10] Kirkland KB, Briggs JP, Trivette SL, Wilkinson WE, Sexton DJ (1999). The impact of surgical-site infections in the 1990s: attributable mortality, excess length of hospitalization, and extra costs. Infect Control Hosp Epidemiol.

[11] Boyce JM, Potter-Bynoe G, Dziobek L (1990). Hospital reimbursement patterns among patients with surgical wound infections following open heart surgery. Infect Control Hosp Epidemiol.

[12] Asensio Vegas A, Monge Jodra V, Lizán García M, Asensio Vegasi A, Lizan Garcia M (1993). Nosocomial infection in surgery wards: a controlled study of increased duration of hospital stays and direct cost of hospitalization. Source Eur J Epidemiol Eur J Epidemiol Eur J Epidemiol.

[13] Poulsen KB, Bremmelgaard A, Sorensen AI, Raahave D, Petersen JV (1994). Estimated costs of postoperative wound infections: a case-control study of marginal hospital and social security costs. Epidemiol Infect.

[14] Kurz A, Sessler DI, Lenhardt R (1996). Perioperative normothermia to reduce the incidence of surgical-wound infection and shorten hospitalization. N Engl J Med.

[15] Harper CM, Andrzejowski JC, Alexander R (2008). NICE and warm. Br J Anaesth.

[16] Cheng D, Martin J (2011). Evidence-based practice and health technology assessment: a call for anesthesiologists to engage in knowledge translation. Can J Anesth Can d’anesthésie..

[17] Graham ID, Logan J, Harrison MB (2006). Lost in knowledge translation: time for a map?. J Contin Educ Heal Prof.

[18] Tricco AC, Cogo E, Ashoor H (2013). Sustainability of knowledge translation interventions in healthcare decision-making: protocol for a scoping review. BMJ Open.

[19] Straus S, Tetroe J, Graham I. Knowledge translation in health care: moving from evidence to practice. London: BMJ Books; 2013.

[20] Rodgers A, Walker N, Schug S (2000). Reduction of postoperative mortality and morbidity with epidural or spinal anaesthesia: results from overview of randomised trials. BMJ.

[21] Landoni G, Rodseth RN, Santini F (2012). Randomized evidence for reduction of perioperative mortality. J Cardiothorac Vasc Anesth.

[22] Arksey H, O’Malley L (2005). Scoping studies: towards a methodological framework. Int J Soc Res Methodol.

[23] Armstrong R, Hall BJ, Doyle J, Waters E (2011). “Scoping the scope” of a Cochrane review. J Public Health (Bangkok).

[24] Rumrill PD, Fitzgerald SM, Merchant WR (2010). Using scoping literature reviews as a means of understanding and interpreting existing literature. Work.

[25] Brien SE, Lorenzetti DL, Lewis S, Kennedy J, Ghali WA (2010). Overview of a formal scoping review on health system report cards. Implement Sci.

[26] Tricco AC, Lillie E, Zarin W (2018). PRISMA Extension for Scoping Reviews (PRISMA-ScR): checklist and explanation. Ann Intern Med.

[27] Levac D, Colquhoun H, O’Brien K (2010). Scoping studies: advancing the methodology. Implement Sci.

[28] Warters R, Szmuk P, Pivalizza E, Gebhard R, Ezri T (2003). Preoperative antibiotic prophylaxis: the role of the anesthesiologist. Anesthesiology.

[29] Healy DA, Clarke Moloney M, McHugh SM, Grace PA, Walsh SR (2014). Remote ischaemic preconditioning as a method for perioperative cardioprotection: concepts, applications and future directions. Int J Surg.

[30] Auriant I, Jallot A, Hervé P (2001). Noninvasive ventilation reduces mortality in acute respiratory failure following lung resection. Am J Respir Crit Care Med.

[31] Sampson M, McGowan J, Cogo E, Grimshaw J, Moher D, Lefebvre C (2009). An evidence-based practice guideline for the peer review of electronic search strategies. J Clin Epidemiol.

[32] McGowan J, Sampson M, Lefebvre C (2010). An Evidence Based Checklist for the Peer Review of Electronic Search Strategies (PRESS EBC). Evid Based Libr Inf Pract.

[33] Khangura S, Konnyu K, Cushman R, Grimshaw J, Moher D (2012). Evidence summaries: the evolution of a rapid review approach. Syst Rev Rev.

[34] Moher D, Squires JE, Kolehmainen N (2009). Preferred Reporting Items for Systematic Reviews and Meta-Analyses: the PRISMA Statement. Ann Intern Med.

[35] Cooper S, Hulley C, Grimley C (2006). Perioperative peripheral parenteral nutrition for patients undergoing esophagectomy for cancer: a pilot study of safety, surgical, and nutritional outcomes. Int J Surg.

[36] Wu G, Liu Z, Wu Z (2006). Perioperative artificial nutrition in malnourished gastrointestinal cancer patients. World J Gastroenterol.

[37] Duncan DG, Duncan DG, Beck SJ, Hood K, Johansen A (2005). Using dietetic assistants to improve the outcome of hip fracture: a randomised controlled trial of nutritional support in an acute trauma ward. Age Ageing.

[38] Bilgin YM (2004). Double-blind, randomized controlled trial on the effect of leukocyte-depleted erythrocyte transfusions in cardiac valve surgery. Circulation.

[39] van de Watering LM, Hermans J, Houbiers JG (1998). Beneficial effects of leukocyte depletion of transfused blood on postoperative complications in patients undergoing cardiac surgery: a randomized clinical trial. Circulation.

[40] Foss NB, Tange Kristensen M, Søe Jensen P, Palm H, Krasheninnikoff M, Kehlet H (2009). The effects of liberal versus restrictive transfusion thresholds on ambulation after hip fracture surgery. Transfusion.

[41] Kosmadakis N, Messaris E, Maris A (2003). Perioperative erythropoietin administration in patients with gastrointestinal tract cancer prospective randomized double-blind study. Annals of surgery.

[42] Lobo SM, Salgado PF, Castillo VG (2000). Effects of maximizing oxygen delivery on morbidity and mortality in high-risk surgical patients. Crit Care Med.

[43] Meyhoff CS, Jorgensen LN, Wetterslev J, Christensen KB, Rasmussen LS (2012). Increased long-term mortality after a high perioperative inspiratory oxygen fraction during abdominal surgery: follow-up of a randomized clinical trial. Anesth Analg.

[44] Antonelli M, Conti G, Bufi M (2000). Noninvasive ventilation for treatment of acute respiratory failure in patients undergoing solid organ transplantation. JAMA.

[45] Zhu GF, Wang DJ, Lui S (2013). Efficacy and safety of noninvasive positive pressure ventilation in the treatment of acute respiratory failure after cardiac surgery. Chin Med J.

[46] Thielmann M, Kottenberg E, Kleinbongard P (2013). Cardioprotective and prognostic effects of remote ischaemic preconditioning in patients undergoing coronary artery bypass surgery: a single-centre randomised, double-blind, controlled trial. Lancet.

[47] Qiu Z, Chen X, Xu M (2009). Evaluation of preoperative intra-aortic balloon pump in coronary patients with severe left ventricular dysfunction undergoing OPCAB surgery: early and mid-term outcomes. J Cardiothorac Surg.

[48] Durmaz I, Yagdi T, Calkavur T (2003). Prophylactic dialysis in patients with renal dysfunction undergoing on-pump coronary artery bypass surgery. Ann Thorac Surg.

[49] Van den Berghe G, Wouters P, Weekers F, et al. Intensive insulin therapy in critically ill patients. N Engl J Med. 2001;345(19) https://v2dis-prod.evidencepartners.com/Generic/getAttachment2.php?id=1414. Accessed 25 Oct 2017. 10.1056/NEJMoa011300 11794168

[50] Landoni G, Pisano A, Lomivorotov V (2017). Randomized evidence for reduction of perioperative mortality: an updated consensus process. J Cardiothorac Vasc Anesth.

[51] McIsaac DI, Wijeysundera DN, Huang A, Bryson GL, van Walraven C (2018). Association of hospital-level neuraxial anesthesia use for hip fracture surgery with outcomes. Anesthesiology.

[52] Neily J, Mills PD, Young-Xu Y, et al. Association between implementation of a medical team training program and surgical mortality. JAMA. 2010;304(15):1693–700. https://www.ncbi.nlm.nih.gov/pubmed/20959579. 10.1001/jama.2010.1506 20959579

[53] Meurisse M, Hamoir E, Defechereux T (1999). Bilateral neck exploration under hypnosedation: a new standard of care in primary hyperparathyroidism?. Ann Surg.

[54] Williamson PR, Altman DG, Bagley H (2017). The COMET handbook: version 1.0. Trials.

[55] Grocott M, Myles P, Moonesinghe R, Boney O (2017). Core Outcome Measures in Perioperative and Anaesthetic Care (COMPAC): Core Outcome Measures in Effectiveness Trials Initiative (COMET).

[56] Shulman M, Myles P (2016). Measuring perioperative outcome. Curr Opin Anaesthesiol.

[57] Myles PS, Grocott MPW, Boney O (2016). Standardizing end points in perioperative trials: towards a core and extended outcome set. Br J Anaesth.

[58] Findley G, National Confidential Enquiry into Patient Outcome and Death (2011). Knowing the risk: a review of the peri-operative care of surgical patients: summary.

[59] McIsaac D, Lavallée LT, van Walraven C (2017). A retrospective assessment of prognostication in 456,685 patients undergoing elective major non-cardiac surgery. Can J Anesth Can d’anesthésie..

[60] Harsten A, Kehlet H, Toksvig-Larsen S (2013). Recovery after total intravenous general anaesthesia or spinal anaesthesia for total knee arthroplasty: a randomized trial^†^. This article is accompanied by editorial IV. Br J Anaesth.

