# Supplementary material for: Anesthesia interventions that alter perioperative mortality: a scoping review
Source: Syst Rev. 2018 Nov 30;7:218. doi: 10.1186/s13643-018-0863-x (PMC6267894; doi:10.1186/s13643-018-0863-x)
Supplement: Supplementary file 1 — Search strategies. (DOCX 14 kb) [file 13643_2018_863_MOESM1_ESM.docx]

## Appendix 1: Search strategies

**MEDLINE**

| 1 | exp Surgical Procedures, Operative/ |
| --- | --- |
| 2 | (surgery or surgical).ab,ti. |
| 3 | ((operative or surgery or surgical) adj3 (anesthesia or anaesthesia)).ab,ti. |
| 4 | or/1-3 |
| 5 | mortality/ or hospital mortality/ or mortality, premature/ or survival rate/ |
| 6 | mortality.ab,ti. |
| 7 | "rates of death".ab,ti. |
| 8 | or/5-7 |
| 9 | perioperative care/ or intraoperative care/ or perioperative nursing/ or postoperative care/ or preoperative care/ |
| 10 | ((perioperative or preoperative or intraoperative or postoperative) adj3 (intervention or therap$ or treatment or procedure or management)).ab,ti. |
| 11 | "outcome and process assessment (health care)"/ or "process assessment (health care)"/ |
| 12 | or/9-11 |
| 13 | ((randomised controlled trial or controlled clinical trial).pt. or randomized.ab. or placebo.ab. or clinical trials as topic.sh. or randomly.ab. or trial.ti.) not (animals not (animals and humans)).sh. |
| 14 | cohort studies/ or prospective studies/ or retrospective studies/ |
| 15 | or/13-14 |
| 16 | 4 and 8 and 12 and 15 |
| 17 | limit 16 to ("all adult (19 plus years)" or "adolescent (13 to 18 years)") |

**Embase**

| 1 | exp surgery/ |
| --- | --- |
| 2 | (surgery or surgical).ti,ab. |
| 3 | ((operative or surgery or surgical) adj3 (anesthesia or anaesthesia)).ti,ab. |
| 4 | or/1-3 |
| 5 | mortality/ or premature mortality/ or surgical mortality/ |
| 6 | mortality.ti,ab. |
| 7 | "rates of death".ti,ab. |
| 8 | or/5-7 |
| 9 | peroperative care/ |
| 10 | perioperative nursing/ |
| 11 | postoperative care/ |
| 12 | preoperative care/ |
| 13 | perioperative period/ |
| 14 | ((perioperative or preoperative or intraoperative or postoperative) adj3 (intervention or therap$ or treatment or procedure or management)).ti,ab. |
| 15 | cohort analysis/ |
| 16 | retrospective study/ |
| 17 | prospective study/ |
| 18 | or/15-17 |
| 19 | "randomized controlled trial (topic)"/ or placebo/ |
| 20 | randomization/ |
| 21 | double blind procedure/ |
| 22 | single blind procedure/ |
| 23 | (random* or sham or placebo*).ti,ab,hw. |
| 24 | ((singl* or doubl*) adj (blind* or dumm* or mask*)).ti,ab,hw. |
| 25 | ((tripl* or trebl*) adj (blind* or dumm* or mask*)).ti,ab,hw. |
| 26 | or/19-25 |
| 27 | 18 or 26 |
| 28 | 9 or 10 or 11 or 12 or 13 or 14 |
| 29 | 4 and 8 and 27 and 28 |
| 30 | limit 29 to (adolescent <13 to 17 years> or adult <18 to 64 years> or aged <65+ years>) |

**CENTRAL and The Cochrane Library**

1 (surgery or surgical).ab,ti.

2 ((operative or surgery or surgical) adj3 (anesthesia or anaesthesia)).ti,ab.

3 or/1-3

4 mortality.ab,ti.

5 "rates of death".ab,ti.

6 or/4-5

7 ((perioperative or preoperative or intraoperative or postoperative) adj3 (intervention or therap$ or treatment or procedure or management)).ti,ab.

8 ((randomised controlled trial or controlled clinical trial).pt. or randomized.ab. or placebo.ab. or clinical trials as topic.sh. or randomly.ab. or trial.ti.) not (animals not (animals and humans)).sh.

9 (random* or sham or placebo*).ti,ab,hw.

10 ((singl* or doubl*) adj (blind* or dumm* or mask*)).ti,ab,hw.

11 ((tripl* or trebl*) adj (blind* or dumm* or mask*)).ti,ab,hw.

12 or/8-11

13 3 and 6 and 7 and 12

**CINAHL**

S15 S3 AND S10 AND S14

S14 S11 OR S12 OR S13

S13 (MH "Clinical Trials+") OR (MH "Nonrandomized Trials") OR (MH "Systematic Review") OR (MH "Meta-Analysis") OR (systematic* N2 review*) OR (systematic* N2 search*) or trial

S12 (MH "Concurrent Prospective Studies") OR (MH "Prospective Studies")

S11 TX observational OR TX cohort OR TX retrospective

S10 S7 OR S8 OR S9

S9 (MH "Intraoperative Complications")

S8 OR (MH "Preoperative Period) OR (MH "Perioperative Period") OR (MH "Postoperative Period")

S7 (MH "Preoperative Care) OR (MH "Perioperative Care") OR (MH "Intraoperative Care) OR (MH "Postoperative Care")

S6 S4 OR S5

S5 TX mortality OR TX death OR TX survival

S4 (MH "Mortality+") OR (MH "Death")

S3 S1 OR S2

S2 TX surgery OR TX surgical OR TX operative

S1 (MH "Surgery+") OR (MH "Surgical Procedures, Operative")
