# Supplementary material for: Anesthesia interventions that alter perioperative mortality: a scoping review
Source: Syst Rev. 2018 Nov 30;7:218. doi: 10.1186/s13643-018-0863-x (PMC6267894; doi:10.1186/s13643-018-0863-x)
Supplement: Supplementary file 2 — Intervention themes and definitions. (DOCX 15 kb) [file 13643_2018_863_MOESM2_ESM.docx]

| **Additional File 2. Intervention themes and definitions** | |
| --- | --- |
| **Intervention theme** | **Definition** |
| Anesthetic technique | The use of a method to “temporarily induce loss of awareness or sensation”, which may or may not include analgesia, paralysis, amnesia or unconsciousness^58^ |
| Blood product transfusion | “An act, process, or instance of transferring” fluid from human blood products into a vein or artery of a person.^59^ |
| Dialysis | “The separation of substances in solution by means of their unequal diffusion through semipermeable membranes.” In our case, usually referring to hemodialysis, which is “the process of removing blood from an artery, purifying it by dialysis, adding vital substances, and returning it to the patient through a vein.”^60^ |
| Glucose control | Management of blood sugar |
| IV Fluids | “Infusion of liquid substances (usually physiologic fluid) directly into a vein”^61^ |
| Medical Device | Use of “a piece of equipment or mechanism designed to serve a special purpose or serve a special function during the operation.”^62^ |
| Monitoring | Intervention used “to watch, keep track of, or check usually for a special purpose.”^63^ |
| Nutrition | Use of “a process of nourishing.”^64^ |
| Pharmacotherapy | Use of one or more pharmaceutical substances in patient care. |
| Physiotherapy | “The treatment of disease, injury, or deformity by physical methods such as massage, heat treatment, and exercise rather than by Pharmacotherapies or surgery.”^65^ |
| Preoperative Procedure | Intervention used “to prepare or manage the patient prior to surgery, which includes both physical and psychological preparation.”^66^ |
| Protocol/guidelines | Implementation of “a detailed plan of a scientific or medical experiment, treatment or procedure.”^67^ |
| Temperature management | Intervention used to monitor or change the patient’s “degree or intensity of heat as expressed according to a definite and comparative scale.”^68^ |
| Testing | Intervention used “to check the quality, performance, or reliability of [something].”^69^ |
| Ventilation | “The act or process of circulating air or gases through the lungs”^70^ |
