# Supplementary material for: Anesthesia interventions that alter perioperative mortality: a scoping review
Source: Syst Rev. 2018 Nov 30;7:218. doi: 10.1186/s13643-018-0863-x (PMC6267894; doi:10.1186/s13643-018-0863-x)
Supplement: Supplementary file 3 — Study and population characteristics. (DOCX 127 kb) [file 13643_2018_863_MOESM3_ESM.docx]

| **Additional File 3. Study and population characteristics (n = 369)** | | | | | | | | | | |
| --- | --- | --- | --- | --- | --- | --- | --- | --- | --- | --- |
| First author, year | Country | Single or Multicenter, n centers, n patients | Type of surgery | Type of anesthesia | Intervention theme | Perioperative phase of intervention | Age (control; intervention groups) | Patient gender (% male) | ASA score (n [%] unless otherwise stated) | Outcome, timing, reported as primary or secondary |
| Abdelmalak, 2013 | USA | Single, 381 | Vascular | General | Pharmacotherapy | Intraoperative | mean: 64, SD: 11; mean: 64, SD: 11; mean: 65, SD: 12; mean: 63, SD: 11 | 52% | ASA score (control; intervention) | All-cause mortality, 1 year, secondary |
| Acikel, 2007 | Turkey | Single, 110 | Cardiac | General | Pharmacotherapy | Preoperative, Intraoperative, Postoperative | mean: 60, SD: 10; mean: 60, SD: 10 | 72% | ASA II: 30, ASA III: 60, ASA IV: 10; ASA II: 24, ASA III: 65, ASA IV: 11; ASA II: 21, ASA III: 68, ASA IV: 11 | Death, in-hospital, secondary |
| Adabag, 2008 | USA | Single, 102 | Cardiac | General | Pharmacotherapy | Preoperative, Postoperative | mean: 72, SD: 9; mean: 70, SD: 9 | 100% | NR | Operative mortality, in-hospital, 30 days, secondary |
| Aguilera, 2013 | Spain | Single, 40 | Orthopedic | Neuraxial | Pharmacotherapy | Intraoperative | mean: 74.9, SD: 7.0; mean: 72.6, SD: 11; mean: 73.8, SD: 6.7; mean: 72.4, SD: 6.6 | NR | NR | Mortality, 60 days, secondary |
| Alexander, 2008 | Multiple | Multi-site, 130, 2996 | Cardiac | General | Pharmacotherapy | Preoperative, Intraoperative, Postoperative | mean: 67 SD: 58-73; mean: 66 SD: 58-73 | NR | ASA I: 0, ASA II: 28, ASA III: 14; ASA I: 2, ASA II: 27, ASA III: 13; ASA I: 1, ASA II: 31, ASA III: 9 | Mortality, in-hospital, 30 days, primary  All-cause mortality, 4 days, secondary  All-cause mortality, 30 days, secondary  Cardiovascular mortality, 4 days, secondary  Cardiovascular mortality, 30 days, secondary |
| Almqvist, 1995 | Sweden | Single, 230 | General | General | Pharmacotherapy | Intraoperative, Postoperative | mean: 32, range: 13-81; mean: 30, range: 13-77 | 63% | NR | Death, 30 days, secondary |
| Amano, 1995 | Japan | Single, 23 | Cardiac | General | Pharmacotherapy | Intraoperative, Postoperative | mean: 54.2, SD: 1.6; mean: 54.5, SD: 1.8 | NR | NR | Mortality, in-hospital, 30 days, secondary |
| Amar, 2001 | USA | Single, 330 | Thoracic | General | Pharmacotherapy | Postoperative | mean: 67, SD: 10; mean: 66, SD: 10 | 54% | NR | Death, 30 days, secondary |
| Anderson, 2003 | UK | Single, 25 | Colorectal | General | Protocol or guidelines implementation | Preoperative, Intraoperative, Postoperative | mean: 68, range: 65-75; mean: 64, range: 55-68 | 44% | NR | Death, in-hospital, secondary |
| Antonelli, 2000 | Italy | Single, 40 | General or thoracic | NR | Ventilation | Postoperative | Mean: 45; 44 | 65% | NR | Rate of fatal complications, in-hospital, primary |
| Arati, 2009 | India | Single, 100 | General | General, neuraxial | Anesthetic technique | Intraoperative | NR | NR | ASA III: 1, Levels aggregated (specify): I and II: 10; ASA III: 1, Levels aggregated (specify): I and II: 13 | Mortality, NR, secondary |
| Aronson, 2008 | USA | Multi-site, 61, 1506 | Cardiac | General | Pharmacotherapy | Preoperative, Intraoperative, Postoperative | mean: 63.9, SD: 11.1; mean: 65, SD: 10.9; mean: 65.3, SD: 11; mean: 55, SD: 28.5 | 73% | NR | Death,  30 days, primary |
| Auriant, 2001 | France | Single, 48 | Thoracic | NR | Ventilation | Postoperative | Mean: 63, SD:9; Mean: 58.9 SD: 10 | NR | NR | Mortality in-hospital and at 120 days, secondary |
| Baguneid, 2001 | UK | Single, 30 | Cardiac | General | Pharmacotherapy | Intraoperative, Postoperative | mean: 63.4, SD: 8.1; mean: 67.7, SD: 9.4 | 73% | NR | Death, NR, NR |
| Barbagallo, 2012 | Italy | Single, 50 | Thoracic | General, regional, neuraxial | Ventilation related | Postoperative | mean: 65, range: 37-76; mean: 69, range: 24-78 | 70% | NR | Mortality, in-hospital, secondary |
| Barzoi, 2000 | Italy | Single, 60 | Oncological | General, neuraxial | Pharmacotherapy | Intraoperative, Postoperative | mean: 60.6; mean: 61.7 | 47% | ASA II: 25 (2-2); Levels aggregated: median (range): CPAP group 2 (2-3) | Mortality, in-hospital, 7 days, secondary |
| Beaulieu, 2010 | Canada | Single, 119 | Cardiac | General | Pharmacotherapy | Intraoperative, Postoperative | mean: 65, SD: 11; mean: 65, SD: 11 | 56% | ASA II: 16, ASA III: 14; ASA II: 18, ASA III: 12; | Death in hospital, in-hospital, secondary  Death, 6 years, secondary |
| Bell, 1992 | UK | Single, 317 | Cardiac | General | Transfusion related | Intraoperative | mean: 59, SD: 10; mean: 60, SD: 9 | NR | NR | Mortality, in-hospital, secondary |
| Bellantone, 1988 | Italy | Single, 87 | Colorectal | General | Nutritional | Preoperative | mean: 55; mean: 58 | 74% | NR | Postoperative mortality rate, in-hospital, NR |
| Bender, 1997 | USA | Single, 104 | Vascular | General | Device | Preoperative, Intraoperative, Postoperative | mean: 65.5, SD: 1.1; mean: 65.3, SD: 1.2 | 66% | NR | Mortality, in-hospital, secondary |
| Benes, 2010 | Czech Republic | Single, 120 | Major abdominal surgery | General, neuraxial | IV Fluids | Intraoperative | mean: 66.32, SD: 8.38; mean: 66.73, SD: 7.88 | 81% | NR | Mortality - Intention to treat, 30 days, secondary  Mortality, secondary, 30 days |
| Benjamin, 2005 | USA | Multi-site, 5, 148 | Cardiac | General | Transfusion related | Intraoperative, Postoperative | mean: 67; mean: 68 | 93% | ASA I: 0, ASA II: 11, ASA III: 40, ASA IV: 9, ASA V: 0; ASA I: 0, ASA II: 14, ASA III: 37, ASA IV: 9, ASA V: 0; | Primary composite endpoint, in-hospital, primary  Death, in-hospital, secondary |
| Berendes, 2003 | Germany | Single, 73 | Cardiac | General, neuraxial | Anesthetic technique | Intraoperative, Postoperative | mean: 59, SD: 12; mean: 61, SD: 11 | 73% | NR | Mortality, in-hospital, 2 year, secondary |
| Berlauk, 1991 | NR | Single, 87 | Vascular | General | Protocol or guidelines implementation | Preoperative, Intraoperative | mean: 65.8, SD: 11.9; mean: 67.8, SD: 12.4; mean: 61.5, SD:13.5 | NR | NR | Death, in-hospital, primary |
| Besogul, 1999 | Turkey | Single, 30 | Cardiac | General | Pharmacotherapy | Preoperative | mean: 35, range: 20-63; mean: 38, range: 21-60 | 23% | NR | Mortality, in-hospital, NR |
| Bestmann, 2007 | Switzerland | Multi-site, 4, 219 | NR | Neuraxial | Pharmacotherapy | Preoperative, Postoperative | mean: 71, SD: 10; mean: 69, SD: 11 | NR | NR | Mortality, 1 year, primary  Event free survival, 1 year, primary |
| Bilgin, 2004 | Netherlands | Multi-site, 2, 474 | Cardiac | General | Transfusion | Intraoperative | Mean: 66.6, SD: 12.5; mean: 65.3, SD:14.7 | 45% | Parsonnet score  0 to 4: 31, 19  5 to 9: 53, 61  10 to 14: 57, 65  15 to 19: 39, 36  20: 57, 56 | Mortality, 90 days, primary  Mortality, in-hospital, secondary |
| Billings, 2012 | USA | Single, 74 | Cardiac | General | Pharmacotherapy | Preoperative, Postoperative | mean: 66.1, SD: 2.1; mean: 64.4, SD: 2.1; mean: 67, SD: 1.7; | 42% | NR | In hospital mortality, in-hospital, secondary |
| Bilotta, 2009 | Italy | Single, 483 | Neurological | General | Pharmacotherapy | Postoperative | mean: 56.9, SD: 12.65; mean: 57.34, SD: 11.94 | 58% | NR | Overall survival at 6 months, 6 months, secondary |
| Bingol, 2005 | Turkey | Single, 40 | Cardiac | General | Pharmacotherapy | Preoperative, Postoperative | mean: 63.8, SD: 6.67; mean: 63.7, SD: 6.22 | 78% | NR | Death, in-hospital, secondary |
| Birdi, 1997 | UK | Single, 300 | Cardiac | General | Temperature | Intraoperative | mean: 59.8, SD: 8.8; mean: 60.4, SD: 8.7; mean: 60.2, SD: 8.9; | 88% | NR | Mortality, in-hospital, NR |
| Bjordahl, 2012 | USA | Single, 185 | Cardiac | General | Pharmacotherapy | Preoperative, Postoperative | mean: 63.0, SD: 12.4; mean: 63.0, SD: 12.4 | 67% | NR | Death, in-hospital, secondary |
| Blum, 2013 | USA | Single, 100 | NR | General | Ventilation related | Intraoperative | NR | 63% | NR | Mortality at 28 days, 28 days, secondary |
| Bode, 1996 | USA | Single, 315 | Vascular | General, neuraxial | Anesthetic technique | Intraoperative | mean: 68, SD: 12; mean: 68, SD: 11; mean: 68, SD: 11; | 80% | Levels aggregated: 4 (3, 4); Levels aggregated: 4 (3, 4) | Mortality, in-hospital, primary |
| Bohm, 2003 | Multiple | Multi-site, 274, 165 | NR | NR | Pharmacotherapy | Preoperative, Postoperative, after discharge from hospital | mean: 63, range: 22-78; mean: 61.4, range: 36-78 | 79% | NR | Mortality, NR, primary |
| Bohner, 2002 | Germany | Single, 204 | Vascular | General | Device | Intraoperative, Postoperative | mean: 64.5, SD: 11.3; mean: 64.1, SD: 12.3 | 81% | NR | Death, in-hospital, secondary |
| Bolliger, 2007 | Switzerland | Multi-site, 2, 141 | Vascular | General | Pharmacotherapy | Preoperative, Postoperative | mean: 68, SD: 9; mean: 67, SD: 10 | 87% | Levels aggregated: 2.85+/-0.44; Levels aggregated: 2.80+-/0.43 | 30-day mortality, 30 days, secondary  Mortality, 1 year, secondary |
| Bonazzi, 2002 | Italy | Single, 100 | Vascular | General | Device | Preoperative, Intraoperative, Postoperative | mean: 68, range: 62-75; mean: 67, range: 63-75 | 100% | NR | In-hospital mortality, in-hospital, secondary |
| Bouza, 2008 | Spain | Single, 690 | Cardiac | General | Device | Intraoperative, Postoperative | mean: 65.0, SD: 12; mean: 65.7, SD: 11.9 | 56% | NR | Mortality, in-hospital, secondary |
| Bove, 2005 | Italy | Single, 80 | Cardiac | General | Pharmacotherapy | Intraoperative, Postoperative | mean: 69, SD: 8.8; mean: 68, SD: 8.2 | 73% | NR | Mortality, in-hospital, secondary |
| Bowley, 2006 | South Africa | Single, 44 | Trauma | General | Transfusion related | Intraoperative | NR | NR | NR | Survival, in-hospital, secondary |
| Boyd, 1993 | UK | Single, 107 | NR | General | Pharmacotherapy | Preoperative, Intraoperative, Postoperative | Median - 72.5, range: 66, 80 (25th, 75th percentiles); Median - 69, range: 61, 77 (25th, 75th percentiles) | 67% | NR | Mortality, in-hospital, primary |
| Bozorgzadeh, 1999 | USA | Single, 300 | Trauma | General | Pharmacotherapy | Preoperative, Intraoperative, Postoperative | mean: 27.5, SD: 10.7; mean: 26.4, SD: 11 | 87% | NR | Postoperative mortality, in-hospital, NR |
| Brackbill, 2007 | USA | Single, 40 | Cardiac | General | Pharmacotherapy | Intraoperative, Postoperative | mean: 62.1, SD: 15.1, range: 36-83; mean: 65.8, SD: 11.8, range: 45-86 | 83% | NR | Mortality in hospital, in-hospital, secondary  Mortality, 30 days, secondary |
| Braga, 2001 | Italy | Single, 257 | Oncological | General | Nutritional | Postoperative | mean: 62.9, SD: 12.4; mean: 64.1, SD: 13.1 | 54% | NR | Mortality, in-hospital, NR |
| Brandstrup, 2003 | Denmark | Multi-site, 8, 141 | Colorectal | General, neuraxial | IV Fluids | Intraoperative, Postoperative | mean: 69, range: 41-88; mean: 64, range: 42-90 | 50% | NR | Death, in-hospital,30 days, secondary |
| Bromley, 1995 | UK | Single, 50 | General | General | Pharmacotherapy | Intraoperative | NR | NR | ASA I: 32, ASA II: 39, ASA III: 1; ASA I: 34, ASA II: 33, ASA III: 2; | Intraoperative death, in-hospital, secondary  Death, 30 days, secondary |
| Bueno, 2004 | Brazil | Single, 50 | Cardiac | General | IV Fluids | Intraoperative | mean: 40.6, SD: 12.8; mean: 45.4, SD: 12.8 | 34% | NR | Hospital mortality, in-hospital, NR |
| Buettner, 2008 | Germany | Single, 80 | Colorectal | General | Monitoring | Intraoperative | mean: 66, range: 40-84; mean: 61, range: 26-100 | 45% | NR | Mortality, in-hospital, NR |
| Burns, 2005 | Canada | Multi-site, 2, 295 | Cardiac | General | Pharmacotherapy | Intraoperative, Postoperative | mean: 69.2, SD: 9.7; mean: 68.9, SD: 8.9 | 79% | Levels aggregated: Mean ASA class II, range I-III; Levels aggregated: Mean ASA class II, range I-III; | Hospital deaths, in-hospital, secondary |
| Calo, 2005 | Italy | Single, 160 | Cardiac | General | Nutritional | Preoperative, Intraoperative, Postoperative | mean: 64.9, range: 9.1; mean: 66.2, SD: 8.0 | 85% | NR | Death, in-hospital, secondary |
| Cao, 2011 | China | Single, 179 | Oncological | General | Glucose control | Intraoperative, Postoperative | mean: 59.4, SD: 7.3; mean: 58.2, SD: 6.3 | 32% | NR | Mortality, 28 days, secondary |
| Cao, 2011 | China | Single, 248 | Oncological | General | Pharmacotherapy | Postoperative | mean: 59.9, SD: 7.6; mean: 58.5, SD: 8.1 | 65% | ASA I: 21, ASA II: 52, ASA III: 14; ASA I: 25, ASA II: 50, ASA III: 17; | Postoperative mortality, in-hospital, 28 days, secondary |
| Caputo, 2009 | UK | Single, 74 | Cardiac | General, neuraxial | Anesthetic technique | Preoperative, Intraoperative, Postoperative | mean: 66.5, SD: 9.3; mean: 63.8, SD: 9.8 | 89% | ASA I: 21, ASA II: 52, ASA III: 14; ASA I: 25, ASA II: 50, ASA III: 17; | Mortality, in-hospital, secondary |
| Caputo, 2011 | Multiple | Multi-site, 2, 691 | Cardiac | General | Pharmacotherapy | Intraoperative | mean: 66, SD: 93.6; mean: 65.5, SD: 8.48 | NR | NR | 30-day mortality, 30 days, NR |
| Carrère, 2007 | France | Single, 84 | General | General | Device | Intraoperative, Postoperative | mean: 66.3, SD: 1.9; mean: 67.1, SD: 1.8 | 50% | NR | Mortality, 30 days, secondary |
| Carrier, 2008 | Multiple | Multi-site, 40, 861 | Cardiac | General | Pharmacotherapy | Preoperative, Intraoperative, Postoperative | mean: 65.7, SD: 9.8; mean: 65.5, SD: 10.9; mean: 65.1, SD: 10.0; | 79% | ASA I: 22, ASA II: 16, ASA III: 3; ASA I: 25, ASA II: 14, ASA III: 4; | Mortality, 90 days, primary |
| Carson, 2011 | Multiple | Multi-site, 47, 1998 | Orthopedic | General, neuraxial | Transfusion related | Intraoperative, Postoperative | mean: 81.8, SD: 8.8; mean: 81.5, SD: 9.0 | 24% | NR | Mortality, 30 days, primary |
| Catena, 2013 | Italy | Multi-site, NR, 142 | General | NR | Pharmacotherapy | Intraoperative, Postoperative | mean: 52.3; mean: 51 | 51% | Levels aggregated: ASA score for Liberal strategy group was 3.0±0.6.; Levels aggregated: ASA score for Restrictive strategy group was 2.9±0.6.; | Mortality, 3 days, secondary |
| Celik, 2008 | Turkey | Single, 50 | Oncological | General | Nutritional | Preoperative, Postoperative | mean: 62.6, SD: 5.4; mean: 63.5, SD: 8.6 | NR | NR | Mortality, NR, NR |
| Chan, 2009 | Brazil | Single, 109 | Cardiac | General | Pharmacotherapy | Intraoperative, Postoperative | mean: 58, SD: 12; mean: 57, SD: 12 | 50% | NR | Survival rate, 30 days, secondary |
| Chaney, 1996 | USA | Single, 40 | Cardiac | General, neuraxial | Pharmacotherapy | Intraoperative | mean: 64, SD: 10; mean: 65, SD: 9 | 93% | ASA IV: 86.3%; ASA IV: 70.2; | Death, NR, secondary |
| Chaney, 1996 | USA | Single, 60 | Cardiac | General, neuraxial | Pharmacotherapy | Intraoperative | mean: 61, SD: 14, range: 37-77; mean: 64, SD: 13, range: 34-80 | 48% | NR | Death, in-hospital, secondary |
| Chang, 2002 | Republic of Korea | Single, 136 | General | General | Device | Preoperative, Intraoperative, Postoperative | mean: 55.3, range: 32-71; mean: 57.8, range: 27-77 | 74% | NR | Mortality, in-hospital, primary |
| Chello, 2006 | Italy | Single, 40 | Cardiac | General | Pharmacotherapy | Preoperative | mean: 63.7, SD: 7.1; mean: 65.7, SD: 7.7 | 78% | NR | Death, in-hospital, secondary |
| Chen, 2007 | USA | Single, 36 | Cardiac | General | Pharmacotherapy | Intraoperative, Postoperative | mean: 78, SD: 7; mean: 77, SD: 10 | 61% | NR | In hospital mortality, in-hospital, secondary |
| Choi, 2013 | Republic of Korea | Single, 100 | Cardiac | General | Pharmacotherapy | Intraoperative, Postoperative | mean: 65, SD: 8, range: 20-75; mean: 63, SD: 9, range: 20-75 | 75% | NR | Death, in-hospital, secondary |
| Cicekcioglu, 2006 | Turkey | Single, 44 | Cardiac | General | Pharmacotherapy | Intraoperative | mean: 48.3, SD: 9; mean: 48.6, SD: 12.1 | 84% | NR | Death, in-hospital, secondary |
| Clinkscale, 2012 | USA | Single, 280 | NR | NR | Device | NR | mean: 50.2, SD: 20; mean: 50.4, SD: 18.6 | 54% | NR | 30-day mortality, 30 days, secondary |
| Closset, 2008 | Belgium | Single, 50 | General | General | Pharmacotherapy | Intraoperative, Postoperative | mean: 65.8, range: 35-80; mean: 66.68, range: 39-82; | 48% | NR | Mortality, 30 days, secondary |
| Coleman, 1989 | USA | Single, 22 | Cardiac | General | Pharmacotherapy | Postoperative | mean: 64, SD: 7; mean: 60, SD: 10 | 68% | NR | 60d mortality, 60 days, secondary |
| Colizza, 1987 | Italy | Single, 52 | Oncological | General | Pharmacotherapy | Intraoperative, Postoperative | mean: 61.9; mean: 61.7 | NR | NR | Operative mortality, NR, secondary |
| Comerota, 1993 | USA | Single, 144 | Vascular | General, neuraxial | Pharmacotherapy | Intraoperative | mean: 63; mean: 64.9; mean: 69.6; mean: 67.1 | 54% | NR | Death, NR, secondary |
| Constantini, 2001 | Israel | Single, 103 | Neurological | General | Pharmacotherapy | Preoperative, Intraoperative, Postoperative | mean: 54, SD: 2; mean: 57.5, SD: 1.5 | 47% | NR | Postoperative mortality, in-hospital, NR |
| Cooper, 2006 | UK | Single, 27 | Oncological | General | Nutritional | Preoperative, Postoperative | mean: 72.5, range: 65-83; mean: 65, range: 47-74 | 81% | NR | 90-day postoperative mortality, 90 days, primary  Postoperative mortality, 1 year, primary |
| Coselli, 2002 | USA | Single, 156 | Vascular | General | Device | Intraoperative | mean: 65.5, SD: 10.9; mean: 65.5, SD: 10.2 | 58% | NR | 30d mortality, 30 days, secondary  Mortality, in-hospital, secondary |
| Crescenti, 2011 | Italy | Single, 200 | Urology | General, neuraxial | Pharmacotherapy | Preoperative, Intraoperative | mean: 64, SD: 7.8; mean: 64, SD: (7.4) | NR | NR | Death, 30 days, NR |
| Dahl, 2010 | Denmark | Single, 99 | Cardiac | General | Pharmacotherapy | Postoperative, after discharge from hospital | mean: 72.6, SD: 10; mean: 72.3, SD: 8 | 72% | Levels aggregated: >2;5; Levels aggregated: >2:8 | Mortality, 1 year, secondary |
| Dalmau, 2000 | Spain | Multi-site, 2, 124 | General | General | Pharmacotherapy | Intraoperative | mean: 60, range: 18-67; mean: 58, range: 22-69; mean: 56, range: 32-69; | 64% | NR | Mortality, 5 months, secondary |
| Dalmau, 2004 | Spain | Single, 127 | General | General | Pharmacotherapy | Intraoperative | mean: 54, SD: 9, range: 22-67; mean: 53, SD: 10, range: 29-68 | 70% | NR | Perioperative mortality, in-hospital, NR |
| Daoud, 2000 | USA | Single, 118 | Cardiac | General | Device | Postoperative | mean: 63, SD: 11; mean: 68, SD: 11; mean: 66, SD: 10; | 60% | NR | Mortality, in-hospital, primary |
| Davies, 2010 | UK | Single, 124 | Major colorectal or urological | General, regional | Pharmacotherapy | Intraoperative, Postoperative | mean: 79, range: 57-89; mean: 74, range: 49-89 | 55% | NR | Mortality, in-hospital, secondary |
| Davies, 1993 | Australia | Single, 50 | Vascular | General, neuraxial, | Anesthetic technique | Intraoperative, Postoperative | mean: 67, SD: 8; mean: 65, SD: 9 | 88% | NR | Mortality, in-hospital, secondary |
| De Luca, 2005 | Italy | Single, 122 | Cardiac | sedation, local | Pharmacotherapy | Intraoperative, Postoperative | mean: 63.6, SD: 10.4; mean: 60.7, SD: 9.8 | 66% | Levels aggregated: Mean ASA for GA group was 2.4±0.6.; Levels aggregated: Mean ASA for CEGA group was 2.5±0.5. | Cardiac mortality, 1 year, secondary |
| De Luis, 2002 | Spain | Single, 47 | Oncological | General | Nutritional | Postoperative | mean: 59.3, SD: 10.5; mean: 63.15, SD: 12.7 | 89% | NR | Mortality, 90 days, primary |
| Desai, 2012 | USA | Single, 189 | Cardiac | General | Glucose control | Postoperative | mean: 62.5, SD: 10.2; mean: 62.8, SD: 9.5 | NR | NR | Mortality, 30 days, secondary |
| Devereaux, 2014 | Multiple | Multi-site, 135, 10010 | Non-cardiac | General, regional, neuraxial | Pharmacotherapy | Preoperative, Postoperative | mean: 68.6, SD: 10.3; mean: 68.6, SD: 10.3 | 53% | NR | Primary composite outcome, 30 days, primary  Death from any cause, 30 days, secondary  Death from cardiovascular cause, 30 days, secondary |
| Devereaux, 2014 | Multiple | Multi-site, 135, 10010 | Non-cardiac | General, regional, neuraxial | Pharmacotherapy | Preoperative, Intraoperative, Postoperative, other: At 2 to 4 hours before surgery, for 72 hours | mean: 68.6, SD: 10.3; mean: 68.5, SD: 10.4 | 53% | NR | Composite of death or nonfatal MI, 30 days, primary  Death, 30 days, secondary  Death from vascular cause, 30 days, secondary |
| Devereaux, 2008 | Multiple | Multi-site, 190, 8331 | Non-cardiac | General, regional, neuraxial | Pharmacotherapy | Preoperative, Postoperative, after discharge from hospital | mean: 69.1, SD: 10.4; mean: 68.9, SD: 10.5 | NR | NR | Cardiovascular death, NR, primary |
| Dieleman, 2012 | Netherlands | Multi-site, 8, 4482 | Cardiac | General | Pharmacotherapy | Intraoperative | mean: 66.1, SD: 10.7; mean: 66.2, SD: 11.0 | 73% | NR | Death, 30 days, primary |
| Diprose, 2005 | UK | Single, 19 | Cardiac | General | Pharmacotherapy | Intraoperative | median: 69.5, range: 63.5-76.5; median: 63, range: 59-66 | NR | NR | Mortality, in-hospital, primary |
| Doglietto, 2004 | Italy | Multi-site, 14, 237 | General | General | Device | Intraoperative, Postoperative | mean: 63.3, SD: 12.2; mean: 62.5, SD: 11.3 | 58% | NR | Mortality, in-hospital, secondary |
| Doglietto, 1996 | Italy | Multi-site, NR,678 | Major abdominal surgery | General | Nutritional | Postoperative | mean: 61, SD: 10.5; mean: 61.1, SD: 10.8 | 58% | NR | Mortality, in-hospital, primary |
| Donati, 2007 | Italy | Multi-site, 9, 135 | Major abdominal surgery | General | Protocol or guidelines implementation | Intraoperative, Postoperative | mean: 66.1, SD: 7.1; mean: 66.0, SD: 7.7 | 65% | NR | Mortality, in-hospital, secondary |
| Donato, 2007 | Italy | Multi-site, 22, 192 | Vascular | NR | Pharmacotherapy | Intraoperative, Postoperative | mean: 81.0, SD: 6.5; mean: 80.3, SD: 6.1 | 51% | ASA II: 11, ASA III: 45, ASA IV: 11; ASA II: 9, ASA III: 49, ASA IV: 10; | Death, 90 days, primary |
| Donato, 2007 | Italy | Multi-site, 22, 300 | Vascular | NR | Pharmacotherapy | Intraoperative, Postoperative | mean: 73.3, SD: 12.1; mean: 74.3, SD: 11.1 | 60% | NR | Mortality, 90 days, primary |
| Dorge, 2000 | Germany | Single, 150 | Cardiac | General | Pharmacotherapy | Intraoperative, Postoperative | mean: 63, SD: 9; mean: 63, SD: 8; mean: 61, SD: 9; | 88% | NR | Hospital mortality, in-hospital, NR |
| Duncan, 2005 | UK | Single, 302 | Orthopedic | NR | Nutritional | Postoperative | NR | 0% | NR | Deaths in trauma unit, in-hospital, primary  Death, 4 months, secondary  Death, in-hospital, secondary |
| Dunkelgrun, 2009* | Netherlands | Multi-site, NR, 1066 | Non-cardiac | General, neuraxial, local | Pharmacotherapy | Preoperative, Intraoperative, Postoperative, after discharge from hospital | mean: 65.6, range: 57-74; mean: 66.8, range: 58-74; mean: 65.4, range: 59-73; mean: 63.8, range: 56-74 | 60% | NR | Cardiac death, in-hospital, 30 days, primary |
| Durazzo, 2004 | Brazil | Single, 100 | Vascular | NR | Pharmacotherapy | Preoperative, Postoperative, after discharge from hospital | mean: 63.38, SD: 9.53, range: 46-83; mean: 65.92, SD: 9.89, range: 36-86 | 79% | ASA I: 96, ASA II: 149, ASA III: 23; ASA I: 95, ASA II: 144, ASA III: 25; ASA I: 96, ASA II: 140, ASA III: 29 | Composite score of major adverse Cardiac events, 6 months, primary  Death from cardiac causes, 6 months, secondary |
| Durmaz, 2003 | Turkey | Single, 44 | Cardiac | General | Dialysis | Preoperative, Postoperative | mean: 54.3, SD: 11.06; mean: 58.1, SD: 11.84 | 80% | NR | In-hospital mortality, in-hospital, 30 days, NR |
| Durukan, 2013 | Turkey | Single, 59 | Cardiac | General | Ventilation related | Intraoperative | mean: 62.48, SD: 6.42; mean: 59.40, SD: 11.10 | 83% | NR | Mortality, in-hospital, secondary |
| Dyke, 2006 | Multiple | Multi-site, 21, 150 | Cardiac | General | Pharmacotherapy | Intraoperative | mean: 65.1, SD: 9.8; mean: 63.8, SD: 11.0 | 56% | NR | 30d mortality, 30 days, secondary  Mortality, 12 weeks, secondary  Mortality, 90 days, secondary |
| El-Tahan, 2010 | Saudi Arabia | Single, 60 | Cardiac | General | Device | Intraoperative | mean: 27.3, SD: 7.8; mean: 28.4, SD: 8.3 | 65% | NR | Mortality, 30 days, secondary |
| Engel, 2009 | Germany | Single, 78 | Cardiac | General | Nutritional | Intraoperative, Postoperative | mean: 69.5, SD: 7.5; mean: 71.3, SD: 7.4; mean: 69.5, SD: 9.1; | 68% | NR | Mortality, in-hospital, secondary |
| Eriksson, 2001 | Multiple | Multi-site, 99, 1250 | Orthopedic | General, regional | Pharmacotherapy | Preoperative, Intraoperative, Postoperative, after discharge from hospital | mean: 67, range: 20-89; mean: 67, range: 24-88 | 84% | Levels aggregated: 3 +/- 0.2; Levels aggregated: 3.1 +/- 0.2; Levels aggregated: 3 +/- 0.2 | Mortality from all causes, 49 days, secondary |
| Eriksson, 2003 | Multiple | Multi-site, 77, 2764 | Orthopedic | General, regional | Pharmacotherapy | Preoperative, Intraoperative, Postoperative, after discharge from hospital | mean: 77.3, SD: 12.6; mean: 76.8, SD: 12.3 | 15% | NR | Death, 6 weeks, primary |
| Eriksson, 2007 | Multiple | Multi-site, 115,3113 | Orthopedic | General, neuraxial | Pharmacotherapy | Postoperative | mean: 64, SD: 11; mean: 65, SD: 10; mean: 63, SD: 11; | NR | NR | Death, in-hospital, 28 days, primary |
| Ersoy, 2013 | Turkey | Single, 20 | Cardiac | General | Pharmacotherapy | Intraoperative, Postoperative | mean: 45.7, SD: 7.9; mean: 49.6, SD: 10.7 | 40% | NR | Mortality, in-hospital, secondary |
| Evonich, 2007 | USA | Single, 40 | Cardiac | General | Device | Intraoperative, Postoperative | mean: 68, SD: 8; mean: 63, SD: 13; mean: 66, SD: 13; | 85% | NR | Mortality, in-hospital, secondary |
| Falcone, 2003 | USA | Single, 99 | Vascular | NR | Testing | Preoperative | mean: 65.5, SD: 11; mean: 66.4, SD: 10 | 68% | NR | In-hospital mortality, in-hospital, secondary  Mortality, 1 year, primary |
| Fan, 1994 | China | Single, 124 | Oncological | General | Nutritional | Preoperative, Postoperative | mean: 53, range: 33-79; mean: 54, range: 28-72 | 88% | NR | Mortality in the hospital, in-hospital, primary |
| Farran, 2008 | Spain | Single, 91 | Oncological | General | Pharmacotherapy | Preoperative, Postoperative | NR | NR | NR | Mortality, in-hospital, primary |
| Fattouch, 2005 | Italy | Single, 58 | Cardiac | General | Pharmacotherapy | Intraoperative | mean: 62, SD: 8; mean: 63, SD: 9; mean: 59, SD: 7 | 45% | NR | Operative mortality, in-hospital, NR  Mortality, in-hospital, NR |
| Fattouch, 2006 | Italy | Single, 58 | Cardiac | General | Pharmacotherapy | Intraoperative | mean: 64, SD: 7; mean: 62, SD: 8; mean: 65, SD: 9; | NR | NR | In hospital mortality, in-hospital, NR |
| Fawzy, 2009 | Saudi Arabia | Single, 38 | Cardiac | General | Pharmacotherapy | Intraoperative | mean: 60, SD: 7; mean: 55, SD: 11 | 95% | NR | Mortality, in-hospital, NR |
| Fergusson, 2008 | Canada | Multi-site, 19, 2331 | Cardiac | General | Pharmacotherapy | Intraoperative | Mean: 67, SD: 10.8; mean: 66.9, SD: 11.4; mean: 66.6, SD:10.8 | 72% | NR | Death from any cause at 30-days, primary |
| Fernandes, 2011 | Brazil | Single, 29 | Cardiac | General | Pharmacotherapy | Intraoperative, Postoperative | mean: 44, SD: 13; mean: 48, SD: 11 | NR | NR | Postoperative mortality, in-hospital, NR |
| Fischer, 2010 | USA | Single, 130 | General | General | Transfusion related | Preoperative, Intraoperative | mean: 68, range: 35-84; mean: 61, range: 44-83 | 53% | NR | Mortality, 90 days, secondary |
| Fleron, 2003 | France | Single, 217 | Vascular | General, neuraxial | Pharmacotherapy | Intraoperative | mean: 66, SD: 10; mean: 67, SD: 11 | 88% | ASA I: 0, ASA II: 46, ASA III: 19; ASA I: 1, ASA II: 47, ASA III: 17; | Mortality, 30 days, NR |
| Fontan, 1992 | France | Single, 120 | Cardiac | General | Protocol or guidelines implementation | Intraoperative | mean: 63, SD: 1.0; mean: 64, SD: 2.5; mean: 63, SD: 1.4; mean: 62, SD: 2.6 | NR | NR | Mortality, 6 days, primary |
| Foss, 2005 | Denmark | Single, 55 | Orthopedic | General, regional | Pharmacotherapy | Postoperative | mean: 81, SD: 7.3; mean: 81, SD: 6.8 | NR | NR | 30-day mortality, 30 days, NR |
| Foss, 2009 | Denmark | Single, 107 | Orthopedic | Neuraxial, sedation | Transfusion related | Intraoperative, Postoperative | mean: 84, SD: 11.4 ; mean: 81, SD: 10.8 | 9% | ASA I: 2, ASA II: 35, ASA III: 23, ASA IV: 0; ASA I: 9, ASA II: 39, ASA III: 12, ASA IV: 0; | 30-day mortality, 30 days, secondary |
| Franke, 2003 | Germany | Single, 200 | Cardiac | General | Pharmacotherapy | Intraoperative | mean: 63.2, SD: 9.6; mean: 65.1, SD: 8.7 | 76% | NR | Operative mortality, in-hospital, 30 days, primary |
| Fremes, 2000 | Canada | Multi-site, 3, 762 | Cardiac | General | Pharmacotherapy | Intraoperative | mean: 60.5, SD: 9.4; mean: 61.4, SD: 8.7 | NR | NR | Late survival, in-hospital, 30 days, 90 days, 1 year, 3 years, 5 years, 7.5 years, primary |
| Friess, 1995 | Germany | Multi-site, 19, 194 | General | General | Pharmacotherapy | Preoperative, Intraoperative, Postoperative | mean: 51, range: 20-80; mean: 52, range: 19-75 | NR | NR | Death, 90 days, secondary  Postoperative complications,90 days, primary |
| Friess, 1994 | Multiple | Multi-site, 18, 322 | General | General | Pharmacotherapy | Preoperative, Intraoperative, Postoperative | mean: 47, range: (20-76); mean: 48, range: 19-72 | 60% | NR | Death, in-hospital, 90 days, primary |
| Futier, 2010 | France | Single, 70 | Major surgery | General | IV Fluids | Intraoperative | mean: 60.4, SD: 14; mean: 61.9, SD: 12 | 55% | NR | Death, NR, secondary |
| Gamberini, 2009 | Switzerland | Single, 113 | Cardiac | General | Pharmacotherapy | Preoperative, Intraoperative, Postoperative | mean: 74.4, SD: 5.9; mean: 74.1, SD: 5.2 | 68% | ASA I: 60, ASA II: 18, ASA III: 22, ASA I: 54, ASA II: 25, ASA III: 21 | Death, in-hospital, secondary |
| Gandhi, 2014 | India | Single, 40 | Cardiac | General | Pharmacotherapy | Preoperative | mean: 39.35, SD: 9.98; mean: 38.20, SD: 7.36 | 50% | NR | Mortality, NR, secondary |
| Gasparovic, 2014 | NR | Single, 219 | Cardiac | Other: antiplatelet and aspirin | Pharmacotherapy | Preoperative | mean: 65, SD: 9; mean: 65, SD: 8 | NR | NR | All-cause death composite, primary |
| Gatt, 2005 | UK | Single, 34 | Colorectal | General, neuraxial | Protocol or guidelines implementation | Preoperative, Intraoperative, Postoperative | mean: 67, range: 60-73; mean: 67, range: 59-76 | 68% | NR | Mortality, NR, NR |
| Ghaffarinejad, 2007 | Iran | Single, 200 | Cardiac | General | Pharmacotherapy | Preoperative | mean: 56.9, SD: 9.14; mean: 56.9, SD: 8.59 | 69% | Levels aggregated: Median (IQ range): Control 2 (2-3); Levels aggregated: Median (IQ range): Control 2 (2-2) | In-hospital mortality, in-hospital, primary |
| Giakoumidakis, 2012 | Greece | Single, 212 | Cardiac | General | Pharmacotherapy | Intraoperative, Postoperative | mean: 66.9, SD: 11.1; mean: 64.9, SD: 11.5 | 67% | NR | In-hospital mortality, in-hospital, primary  Mortality, 30 days, primary |
| Giger-Pabst, 2013 | Switzerland | Multi-site, 6, 108 | Oncological | General | Nutritional | Preoperative | mean: 63.2, SD: 11.8; mean: 64.9, SD: 13.6 | 61% | NR | Mortality, in-hospital, 30 days, NR |
| Giri, 2001 | USA | Single, 220 | Cardiac | General | Pharmacotherapy | Preoperative, Intraoperative, Postoperative | mean: 72.5, SD: 6.7; mean: 72.7, SD: 6.7 | 76% | NR | Mortality, 30 days, secondary |
| Goeters, 2002 | Germany | Single, 95 | NR | NR | Nutritional | Postoperative | mean: 53.1, SD: 17.3; mean: 50.2, SD: 17.8 | 65% | NR | 6-month mortality, 6 months, NR  Mortality, 30 days, NR  Mortality, in-hospital, NR |
| Gomez-Hernandez, 2010 | Mexico | Single, 70 | General | General | Pharmacotherapy | Intraoperative | mean: 49.89, SD: 10.58; mean: 50.11, SD: 12.37 | 0% | NR | Mortality, NR, NR |
| Gonenc, 2014 | Turkey | Single, 47 | General | General | Protocol or guidelines implementation | Postoperative | mean: 37.8, SD: 14.3, range: 18-71; mean: 35.4, SD: 13.2, range: 18-66 | 77% | NR | Mortality, 30 days, primary |
| Goodrich, 1993 | USA | Single, 64 | General | General | Pharmacotherapy | Preoperative, Intraoperative, Postoperative | mean: 34.3, range: 4-57; mean: 34.5, range: 5-55 | 52% | NR | Mortality, 100 days, secondary  Death, 180 days, secondary |
| Gordon, 1965 | USA | Single, 191 | Colorectal | NR | Pharmacotherapy | Preoperative | NR | NR | NR | Death, NR, secondary  Death, NR, secondary |
| Gordon, 1991 | USA | Single, 395 | General | General | Nutritional | Preoperative, Postoperative | NR | NR | NR | 30- and 90- day mortality, 30 days, 60 days, 90 days, secondary |
| Grande, 1996 | Spain | Single, 63 | General | General | Device | Intraoperative | mean: 50, range: 32-60; mean: 53, range: 21-59 | 70% | NR | Early post-operative mortality, 30 days, secondary |
| Guarnieri, 1999 | USA | Single, 300 | Cardiac | General | Pharmacotherapy | Postoperative | mean: 63.9; mean: 63.3 | NR | NR | Mortality, 30 days, secondary |
| Güden, 2002 | Turkey | Single, 60 | Cardiac | General | Pharmacotherapy | Intraoperative | mean: 66, SD: 10; mean: 64, SD: 5 | 62% | NR | Mortality, NR, secondary |
| Gunjan, 2007 | USA | Single, 371 | Cardiac | General | Pharmacotherapy | Intraoperative | mean: 63, SD: 16; mean: 63, SD: 15 | 69% | NR | Primary composition endpoint, 30 days, primary  Death, in-hospital, secondary  Death, 30 days, secondary |
| Haase, 2013 | Multiple | Multi-site, 4,350 | Cardiac | NR | Pharmacotherapy | Intraoperative | mean: 64.6, SD: 13.5; mean: 66.4, SD: 12.1 | 71% | ASA II: 2, ASA III: 163, ASA IV: 20; ASA II: 3, ASA III: 161, ASA IV: 19; | Died in hospital, in-hospital, secondary  Died after discharge, 90 days, secondary  Overall mortality, 90 days, secondary |
| Hajjar, 2010 | Brazil | Single, 502 | Cardiac | General | Transfusion related | Intraoperative, Postoperative | mean: 60.7, SD: 12.5; mean: 58.6, SD: 12.5 | 62% | NR | Operative Mortality, 30 days, primary |
| Hamaji, 2013 | Brazil | Single, 48 | Orthopedic | neuraxial | Pharmacotherapy | Preoperative | mean: 53, range: 47-59; mean: 54, range: 47-61 | 48% | NR | Death, in-hospital, secondary |
| Han, 2012 | Taiwan | Single, 30 | Major surgery | NR | Nutritional | Postoperative | range: 19-63; range: 34-74 | 87% | NR | Mortality, in-hospital, secondary |
| Hassanain, 2013 | Canada | Single, 56 | Oncological | General, neuraxial | Pharmacotherapy, Glucose | Preoperative, Intraoperative, Postoperative | NR | NR | NR | Mortality, 30 days, secondary |
| Haussen, 1992 | Germany | Single, 41 | Cardiac | General | Pharmacotherapy | Postoperative | mean: 61, SD: 7; mean: 57, SD: 9; mean: 56, SD: 12; | 32% | NR | Postoperative mortality, in-hospital, NR  Late mortality, 6 months, NR |
| Hayashi, 2011 | Japan | Single, 200 | General | General | Pharmacotherapy | Intraoperative, Postoperative | mean: 70, range: 35-82; mean: 69, range: 39-81 | NR | NR | Perioperative mortality, in-hospital, 30 days, secondary |
| Haynes, 2002 | UK | Multi-site, 8, 145 | Vascular | General | Transfusion related | Intraoperative | NR | NR | NR | Death, in-hospital, primary |
| Hecht-Dolnik, 2009 | USA | Single, 156 | Cardiac | General | Pharmacotherapy | Intraoperative | mean: 64.4, SD: 9.0; mean: 64.1, SD: 9.8 | 100% | NR | Mortality in-hospital, secondary |
| Heidari, 2011 | Iran | Single, 400 | Orthopedic | General, neuraxial | Anesthestic technique | Intraoperative | GA: >40 – 75.4%; NA: >40 – 76.9% | 64% | NR | Mortality, in-hospital and out of hospital, NR |
| Hekmat, 2004 | Germany | Single, 118 | Cardiac | General | Pharmacotherapy | Intraoperative | mean: 63, SD: 8; mean: 63, SD: 8 | 86% | NR | Mortality, in-hospital, secondary |
| Helminen, 2007 | Finland | Single, 100 | General | General, neuraxial | Nutritional | Preoperative, Postoperative | mean: 63, SD: 15; mean: 58, SD: 17 | 63% | NR | Mortality, in-hospital, secondary |
| Hemelrijck, 2014 | Multiple | Multi-site, 21, 160 | Non-cardiac | General | Transfusion related | Postoperative | mean: 60.9, SD: 14, range: 22-85; mean: 61.1, SD: 13.2, range: 21-86 | 56% | NR | 30-day mortality, 30 days, secondary |
| Hempenius, 2013 | Netherlands | Multi-site, 3, 260 | General | NR | Protocol or guidelines implementation, Training, education, teamwork | Preoperative, Postoperative | mean: 77.63, SD: 7.69; mean: 77.45, SD: 6.72 | NR | NR | Mortality, in-hospital, secondary |
| Herr, 2000 | USA | Multi-site, 7, 113 | NR | Sedation | Pharmacotherapy | Intraoperative | mean: 59.7, SD: 14.4, range: 17-83; mean: 61.5, SD: 15.5, range: 19-84 | 90% | NR | 7-day mortality, 7 days, primary  Mortality, 28 days, primary |
| Hesse, 2005 | Belgium | Single, 105 | General | General | Pharmacotherapy | Preoperative, Postoperative | mean: 58.98, SD: 13.7; mean: 59.93, SD: 13 | 73% | NR | Death, 30 days, secondary |
| Hirokawa, 2013 | Japan | Single, 188 | General | General | Pharmacotherapy | Postoperative | mean: 68, range: 35-82; mean: 68, range: 22-88 | 66% | NR | Mortality, in-hospital, secondary |
| Hogue, 2007 | USA | Multi-site, 3, 174 | Cardiac | General | Pharmacotherapy | Preoperative, Intraoperative, Postoperative | mean: 71.2, SD: 8.2; mean: 69.6, SD: 9.1 | 0% | ASA I: 45, Levels aggregated (specify) : 49 patients total were ASA≥II; ASA I: 46, Levels aggregated (specify) : 48 patients total were ASA≥II; | 1-month mortality, 1 month, secondary  Mortality, 6 months, NR |
| Höhn, 2002 | Switzerland | Single, 77 | Cardiac | General | Transfusion related | Intraoperative | mean: 62, SD: 12; mean: 65, SD: 10 | 78% | NR | Mortality, in-hospital, secondary |
| Hoogwerf, 1999 | USA | Multi-site, 7, 1351 | Cardiac | General | Pharmacotherapy | Postoperative, after discharge from hospital | mean: 61.4; mean: 63.1 | NR | NR | Death, 5 years, secondary |
| Hourlier, 2014 | France | Single, 164 | Orthopedic | General, regional | Pharmacotherapy | Intraoperative | mean: 68.4, SD: 11.1; mean: 68.7, SD: 9.5 | NR | NR | Death, 90 days, secondary |
| Hsu, 2007 | China | Single, 151 | Oncological | General, neuraxial | Device | Intraoperative, Postoperative | mean: 53.0, SD: 9.31; mean: 57.8, SD: 8.91 | 55% | NR | Mortality, 30 days, secondary |
| Iliuta, 2014 | Romania | Single, 527 | Cardiac | General | Pharmacotherapy | Preoperative, Intraoperative, Postoperative | mean: 63, SD: 13; mean: 63, SD: 12 | 67% | NR | 30-day mortality, 30 days, primary |
| Iliuta, 2009 | Romania | Multi-site, 6, 1352 | Cardiac | General | Pharmacotherapy | Preoperative, Postoperative | mean: 63, SD: 12; mean: 63, SD: 12; mean: 63, SD: 13; | NR | NR | Death at 30 days, 30 days, primary |
| Iliuta, 2003 | Romania | Single, 400 | Cardiac | General | Pharmacotherapy | Postoperative | Mean: 51, SD: 13; Mean: 51, SD: 12 | 37% | NR | Death at 30-days, primary |
| Isgro, 2002 | Germany | Single, 97 | Cardiac | General | Pharmacotherapy | Intraoperative | mean: 66, SD: 7; mean: 64, SD: 9 | 65% | ASA I: 0, ASA II: 1, ASA III: 14, ASA IV: 12; ASA I: 0, ASA II: 2, ASA III: 18, ASA IV: 8; | Mortality, NR, secondary |
| Jebeli, 2010 | Iran | Single, 70 | Cardiac | General | Pharmacotherapy | Intraoperative | mean: 58.2, SD: 8.4; mean: 56.9, SD: 9.7 | 76% | NR | Postoperative death, 1 day, NR |
| Jeong, 2008 | Republic of Korea | Single, 28 | Cardiac | General | Pharmacotherapy | Intraoperative | mean: 55.1, SD: 14.0; mean: 61.2, SD: 11.3 | 64% | NR | Mortality, NR, secondary |
| Jia Shi, 2013 | China | Multi-site, 7, 552 | Cardiac | General | Pharmacotherapy | Intraoperative | mean: 59.6, SD: 9.02; mean: 60, SD: 9.41; mean: 59.9, SD: 9.12; mean: 61.5, SD: 9.19 | 72% | NR | Mortality at discharge, in-hospital, secondary |
| Jones, 2013 | UK | Single, 91 | General | General, neuraxial | Protocol or guidelines implementation | Preoperative, Postoperative | mean: 67, range: 27-84; mean: 64, range: 27-83 | 59% | NR | Mortality, 30 days, secondary |
| Juelsgaard, 1998 | Denmark | Single, 43 | Orthopedic | General, neuraxial | Anesthetic technique | Intraoperative | mean: 79.6, range: 72-92; mean: 85.7, range: 72-94; mean: 82.2, range: 65-99; | 19% | ASA I: 2, ASA II: 38, ASA III: 5; ASA I: 0, ASA II: 43, ASA III: 3; | Cumulative mortality after 1 week,7 days, secondary  Cumulative mortality, 30 days, secondary |
| Juul, 2006 | Denmark | Multi-site, 13, 921 | Non-cardiac | NR | Pharmacotherapy | Preoperative, Intraoperative, Postoperative | mean: 64.8, SD: 11.8; mean: 64.9, SD: 11.1 | NR | ASA II: 9, ASA III: 5, ASA IV: 0; ASA II: 7, ASA III: 6, ASA IV: 1; ASA II: 8, ASA III: 7, ASA IV: 0 | All-cause mortality, 6 months, secondary |
| Kasprzak, 2006 | Germany | Single, 186 | Vascular | General, regional | Anesthetic technique | Intraoperative | mean: 69, SD: 8, range: 46-87; mean: 69, SD: 8, range: 48-84 | 64% | NR | Death related to cardio-pulmonary complications, 30 days, primary  Death due to other causes, 30 days, primary |
| Kaste, 1979 | Finland | Single, 64 | Neurological | NR | Pharmacotherapy | Preoperative | NR | 47% | NR | Mortality, in-hospital, NR |
| Katsourakis, 2013 | Greece | Single, 67 | General | General | Pharmacotherapy | Preoperative, Intraoperative, Postoperative | mean: 61.3, range: 41-83; mean: 62.7, range: 37-84 | 58% | NR | Mortality, 6 months, secondary |
| Katsourakis, 2010 | Greece | Single, 71 | General | General | Pharmacotherapy | Preoperative, Intraoperative, Postoperative | mean: 61.3, range: 41-83; mean: 62.7, range: 37-84 | 55% | NR | Mortality, 90 days, secondary |
| Kerr, 2012 | USA | Single, 98 | Thoracic | General | Pharmacotherapy | Intraoperative, Postoperative | mean: 47, SD: 14; mean: 51, SD: 13 | 46% | NR | Mortality, in-hospital, secondary |
| Kettelhack, 1998 | Germany | Multi-site, 16, 102 | Colorectal | General | Transfusion related | Preoperative, Intraoperative, Postoperative | mean: 67, range: 37-91; mean: 71, range: 53-87 | 42% | NR | Mortality, in-hospital, secondary |
| Khoo, 2007 | UK | Single, 70 | Colorectal | General, neuraxial | Protocol or guidelines implementation | Preoperative, Intraoperative, Postoperative | mean: Median 73.0, range: 46.4-84.6; mean: Median 69.3, range: 46.3-87.7 | 39% | NR | Mortality, in-hospital, 14 days, secondary |
| Kipfer, 2003 | Switzerland | Single, 30 | Cardiac | General | Pharmacotherapy | Intraoperative | mean: 61.3, SD: 7; mean: 62.3, SD: 7 | 83% | ASA I: 3, ASA II: 27, ASA III: 5; ASA I: 5, ASA II: 25, ASA III: 5; | Mortality, NR, secondary |
| Kirdemir, 2008 | Turkey | Multi-site, 2, 200 | Cardiac | General | Pharmacotherapy | Intraoperative, Postoperative | mean: 57, SD: 12; mean: 58, SD: 9 | 62% | NR | Mortality, in-hospital, secondary |
| Kiziltepe, 2004 | Turkey | Single, 40 | Cardiac | General | Pharmacotherapy | Intraoperative | mean: 60.1, SD: 7.9, range: 44-77; mean: 60.3, SD: 13.8, range: 27-78 | 68% | NR | Mortality, in-hospital, secondary |
| Klek, 2008 | Poland | Single, 205 | General | General | Nutritional | Postoperative | mean: 61.4, SD: 11.9; mean: 61.2, SD: 11.7; mean: 60, SD: 11.5; mean: 61.4, SD: 11.8 | 70% | NR | Postoperative mortality, in-hospital, secondary |
| Klemperer, 1995 | USA | Single, 142 | Cardiac | General | Pharmacotherapy | Intraoperative, Postoperative | mean: 68, SD: 9; mean: 66, SD: 10 | 85% | ASA I: 37, ASA II: 16; ASA I: 34, ASA II: 18; ASA I: 33, ASA II: 16 | Mortality, in-hospital, 30 days, secondary |
| Kollef, 1999 | USA | Single, 343 | Cardiac | General | Ventilation related | Postoperative | mean: 62.5, SD: 13.1; mean: 64.7, SD: 12.3 | 65% | NR | Hospital mortality, in-hospital, secondary |
| Korte, 2009 | Switzerland | Single, 22 | General | General | Pharmacotherapy | Intraoperative | NR | NR | NR | Mortality, 1 year, secondary |
| Kosmadakis, 2003 | Greece | Single, 63 | Oncological | NR | Transfusion related | Preoperative, Postoperative | mean: 66.4, SD: 2; mean: 67.1, SD: 2.1 | 54% | NR | Mortality, 1 year, NR |
| Kramer, 2002 | Germany | Single, 56 | Cardiac | General | Pharmacotherapy | Intraoperative, Postoperative | mean: 60.3, SD: 8.1; mean: 60.4, SD: 10.1 | 75% | NR | Mortality, in-hospital, 30 days, secondary |
| Krestchmer, 1989 | Austria | Single, 252 | Vascular | General | Pharmacotherapy | Preoperative, Postoperative, after discharge from hospital | NR | 19% | NR | Probability of survival, 6 years, primary |
| Krishnan, 2014 | Australia | Single, 81 | General | General, local | Pharmacotherapy | Postoperative | mean: 64.5, SD: 19.4; mean: 65.5, SD: 11.0; mean: 53.8, SD: 11.8; mean: 65.5, SD: 11.1 | 56% | NR | Death, 30 days, secondary |
| Kristeller, 2012 | USA | Single, 92 | Cardiac | General | Pharmacotherapy | Preoperative, Intraoperative, Postoperative | mean: 73, SD: 11; mean: 72, SD: 11 | NR | ASA I: 2, ASA II: 0, ASA III: 4; ASA I: 1, ASA II: 10, ASA III: 13; ASA I: 2, ASA II: 14, ASA III: 4 | Mortality, 30 days, secondary |
| Kunstyr, 2008 | Czech Republic | Single, 32 | Thoracic | General, neuraxial | Anesthetic technique | Intraoperative | mean: 46.6, SD: 13.8; mean: 60, SD: 11.1 | 66% | NR | Mortality, in-hospital, secondary |
| Lafere, 2013 | Belgium | Multi-site, 2, 80 | Orthopedic | General, neuraxial | FiO2 | Postoperative | mean: 81.7, SD: 8.41; mean: 80.4, SD: 7.43 | 53% | NR | Mortality, 6 months, NR |
| Laiq, 2013 | Pakistan | Single, 100 | Cardiac | General | Pharmacotherapy | Preoperative, Intraoperative | mean: 40, SD: 11.35, range: 20-60; mean: 36, SD: 12.9, range: 20-60 | 38% | Levels aggregated: All patients were ASAI or ASAII; Levels aggregated: All patients were ASAI or ASAII; | Mortality, in-hospital, secondary |
| Landoni, 2014 | NR | Multi-site, 2, 200 | Cardiac | General | Pharmacotherapy | Intraoperative | mean: 70, range: 50-90; mean: 68, range: 24-84 | 68% | NR | Death during the first hospital stay and/or prolonged ICU stay, in-hospital, primary (composite outcome) |
| Lassen, 2008 | Norway | Multi-site, 5, 447 | General | General, neuraxial | Nutritional | Postoperative | mean: 65, SD: 13.3; mean: 63, SD: 14.4 | 71% | NR | 30-day mortality, 30 days, primary  Total mortality, in-hospital, primary  Total mortality, 8 weeks, primary |
| Lassen, 2002 | Multiple | Multi-site, 73, 2273 | Orthopedic | General, neuraxial | Pharmacotherapy | Preoperative, Postoperative | mean: 67, range: 24-97; mean: 66, range: 29-92 | 42% | NR | Death, 49 days, secondary |
| Launo, 2003 | Italy | Single, 28 | Thoracic | General, neuraxial | Pharmacotherapy | Intraoperative | NR | NR | NR | 30d mortality, 30 days, secondary |
| Lavu, 2014 | USA | Single, 259 | General | General | Pharmacotherapy | Intraoperative | mean: 68.3, range: 25-91; mean: 66.6, range: 25-91 | NR | NR | 30d mortality, 30 days, primary  Mortality, 90 days, primary |
| Lefere, 2013 | Multiple | Multi-site, 2, 80 | Orthopedic | NR | Ventilation related | Postoperative | mean: 81.7, SD: 8.41; mean: 80.4, SD: 7.43 | 48% | NR | 6-month mortality, 6 months, secondary |
| Levin, 2008 | Argentina | Multi-site, 2, 137 | Cardiac | General | Pharmacotherapy | Intraoperative, Postoperative | mean: 62.9; mean: 63.7 | NR | Levels aggregated: 1-2; Levels aggregated: 1-2 | Mortality, 30 days, primary |
| Levin, 2012 | USA | Multi-site, 2, 252 | Cardiac | General | Pharmacotherapy | Preoperative | mean: 61.7; mean: 62.4 | NR | NR | 30-day mortality, 30 days, primary |
| Li, 2006 | China | Single, 93 | Cardiac | General | Glucose control | Postoperative | mean: 63.7; mean: 63.5 | 62% | NR | Mortality, NR, secondary |
| Limberi, 2003 | Greece | Single, 50 | General | General, neuraxial | Anesthetic technique | Intraoperative | mean: 65, SD: 8; mean: 64, SD: 12 | 38% | NR | Mortality, 2 days, secondary |
| Liu, 2010 | China | Single, 63 | Oncological | General | Protocol or guidelines implementation | Preoperative, Intraoperative, Postoperative | mean: 61.9, SD: 8.3; mean: 60.7, SD: 9.7 | 52% | ASA II: 10, ASA III: 15; ASA II: 11, ASA III: 14 | 30d mortality, 30 days, secondary |
| Lobo, 2000 | Brazil | Single, 37 | Oncological | General | Ventilation related, FiO2 | Intraoperative, Postoperative | mean: 63, range: 45-93; mean: 63, range: 45-81 | 65% | Levels aggregated: median 2, range 2-2; Levels aggregated: Median 2, range 2-3 | 28d mortality, 28 days, primary  Mortality, 60 days, primary |
| Lobo, 2006 | Brazil | Single, 50 | General | General | Pharmacotherapy | Intraoperative, Postoperative | mean: 69.9, SD: 8.4; mean: 67.6, SD: 7.5 | 72% | NR | Mortality, in-hospital, 60 days, 28 days, primary |
| Lodge, 2005 | Multiple | Multi-site, NR, 182 | General | General | Pharmacotherapy | Intraoperative | mean: 52.3, SD: 11.5; mean: 53.3, SD: 11.2; mean: 52.6, SD: 9.2; | 60% | NR | Death, in-hospital, secondary |
| Lomivorotov, 2012 | Russia | Single, 90 | Cardiac | General | Pharmacotherapy | Preoperative, Intraoperative, Postoperative | mean: 56.8, SD: 9.4; mean: 57.7, SD: 7.3; mean: 57.3, SD: 8.6; | 91% | NR | Mortality, in-hospital, 30 days, secondary |
| Lorut, 2014 | France | Multi-site, 7, 360 | Thoracic | General, neuraxial | Ventilation related | Postoperative | mean: 63.7, SD: 8.8; mean: 63.6, SD: 10.6 | 77% | NR | Mortality, in-hospital, 30 days, secondary |
| Lu, 2014 | NR | Single, 64 | NR | other: recombinant FVIIa | Pharmacotherapy | Preoperative | mean: 48.7, SD: 12.1; mean: 46.7, SD: 11.2 | 69% | Levels aggregated: ASA III + IV, n (%): Control group 80 (44.6); Levels aggregated: ASA III + IV, n (%): Prophylactic NIV group 67 (37) | All-cause mortality, NR, secondary |
| Lu, 2014 | China | Single, 297 | General | General | Protocol or guidelines implementation | Intraoperative, Postoperative | mean: 52.6, SD: 11.3; mean: 54, SD: 11.4 | 82% | NR | Postoperative mortality, 30 days, secondary |
| MacFie, 2000 | UK | Single, 100 | General | General | Nutritional | Preoperative, Postoperative | mean: 64, range: 42-85; mean: 63, range: 41-86; mean: 68, range: 23-84; mean: 66, range: 23-86 | 46% | ASA I: 88, ASA II: 74; ASA I: 71, ASA II: 64 | Mortality, in-hospital, 30 days, 6 months, secondary |
| Mangano, 2006 | Multiple | Multi-site, 56, 2695 | Cardiac | General | Pharmacotherapy | Intraoperative, Postoperative | mean: 63.2, SD: 9.5; mean: 63.1, SD: 9.6 | 80% | NR | 2-year mortality, 2 year, primary  Mortality in patients with MI, 2 years, secondary |
| Marandola, 2008 | Italy | Single, 40 | General | General, neuraxial | Pharmacotherapy | Intraoperative, Postoperative | mean: 62, SD: 9; mean: 64, SD: 8 | 60% | NR | Operative mortality, in-hospital, secondary |
| Marelich, 2000 | USA | Single, 335 | NR | NR | Protocol or guidelines implementation | Postoperative, other: Not all patients perioperative; used throughout ICU admission | mean: 56.6, SD: 16; mean: 54.5, SD: 17.1; mean: 41, SD: 17.6; mean: 41.5, SD: 18.3 | 66% | ASA I: 6, ASA II: 14, ASA III: 4; ASA I: 4, ASA II: 10, ASA III: 2 | All-cause mortality, in-hospital, secondary |
| Mares, 2000 | Multiple | Multi-site, 2,47 | Cardiac | General | Protocol or guidelines implementation | Intraoperative, Postoperative | mean: 52.2, SD: 2.7, range: 36-67; mean: 54.2, SD: 2.5, range: 19-76 | NR | NR | 30-day mortality, 30 days, NR |
| Martin, 1992 | USA | Single, 177 | General | NR | Pharmacotherapy | Preoperative | mean: 33, SD: 13; mean: 29, SD: 9 | NR | NR | Preoperative survival, in-hospital, primary  Overall survival, in-hospital, primary |
| Matos, 2012 | Brazil | Single, 90 | Cardiac | General | Nutritional | Preoperative, Postoperative | mean: 65.06, SD: 9.81; mean: 63.06, SD: 9.3 | NR | NR | Operative mortality, in-hospital, 30 days, NR |
| McFalls, 2004 | USA | Multi-site, 18, 510 | Cardiac | NR | Preoperative procedure/ surgery | Preoperative | mean: 67.2, SD: 10.4; mean: 65.6, SD: 11.1 | NR | NR | Death within 30 days, 30 days, secondary  Long-term survival, 3 years, primary |
| McGuinness, 2013 | Multiple | Multi-site, 3, 427 | Cardiac | General | Pharmacotherapy | Intraoperative, Postoperative | mean: 67.8, SD: 12.8; mean: 66.3, SD: 14 | 67% | NR | Mortality, in-hospital,90 days, secondary |
| Mentzer, 2007 | USA | Multi-site, 54, 272 | Cardiac | General | Pharmacotherapy | Intraoperative | NR | 73% | NR | 30-day morality, 30 days, secondary  Mortality, 6 months, secondary |
| Mentzer, 2008 | Multiple | Multi-site, 235, 5761 | Cardiac | General | Pharmacotherapy | Intraoperative, Postoperative | mean: 64.1, SD: 11.3; mean: 63.6, SD: 10.5 | 4% | NR | Mortality 5 day, 5 days, primary  Mortality, 30 days, primary  Mortality, 6 months, primary |
| Mentzer, 1999 | Canada, USA | Multi-site, NR, 278 | Cardiac | General |  |  | NR | NR | NR | Death rate, in-hospital, secondary  Death rate, 6 weeks, secondary |
| Mertes, 2006 | Multiple | Multi-site, 18, 199 | General or thoracic | General | Nutritional | Postoperative | mean: 60.2, SD: 13.8; mean: 60.5, SD: 14.2 | 62% | NR | Mortality, in-hospital, secondary |
| Meyhoff, 2012 | Denmark | Multi-site, 14, 1382 | General | General | Ventilation related | Intraoperative, Postoperative | mean: 64, range: 27-85; mean: 64, range: 34-84 | 42% | NR | Mortality, 2 year, primary  Mortality in cancer patient, 2 years, NR  Mortality in non-cancer patient, 2 years, NR |
| Meyhoff, 2009 | Denmark | Multi-site, 14, 1386 | General | General, neuraxial | Ventilation related, FiO2 | Intraoperative, Postoperative | mean: 64, range: 34-84; mean: 64, range: 27-85 | 42% | ASA I: 176, ASA II: 375, ASA III: 130, ASA IV: 4; ASA I: 197, ASA II: 374, ASA III: 125, ASA IV: 5; | 30-day mortality, 30 days, secondary |
| Mitchell, 2005 | Canada | Single, 601 | Cardiac | General | Pharmacotherapy | Preoperative, Postoperative | mean: 61.9, SD: 11.2; mean: 61.3, SD: 11.3 | 82% | ASA I: 199, ASA II: 376, ASA III: 125, ASA IV: 5; ASA I: 176, ASA II: 378, ASA III: 132, ASA IV: 4 | 1 year mortality, 1 year, secondary |
| Moesgaard, 1998 | Denmark | Multi-site, 5, 164 | Colorectal | General | Pharmacotherapy | Intraoperative, Postoperative | mean: 69, range: 18-79; mean: 68, range: 18-82 | 42% | NR | Mortality, in-hospital, 30 days, secondary |
| Molin, 1979 | Sweden | Single, 141 | Colorectal | NR | Pharmacotherapy | Preoperative | NR | 43% | NR | Mortality, in-hospital, NR |
| Mori, 2014 | Japan | Single, 42 | Cardiac | General | Pharmacotherapy | Intraoperative, Postoperative | mean: 73, range: 53-86; mean: 75.5, range: 36-87 | 67% | NR | Mortality, 30 days, secondary |
| Muehling, 2007 | Germany | Single, 55 | Thoracic | General, regional, neuraxial | Protocol or guidelines implementation | Preoperative, Intraoperative, Postoperative | mean: 64, range: 24-83; mean: 67, range: 45-81 | 78% | NR | Death, NR, NR |
| Muehling, 2008 | Germany | Single, 79 | Vascular | General, neuraxial | Protocol or guidelines implementation | Preoperative, Intraoperative, Postoperative | mean: 68, range: 52-84; mean: 67, range: 40-81 | 92% | ASA II: 5, ASA III: 23, ASA IV: 2; ASA II: 4, ASA III: 24, ASA IV: 2 | Death, NR, primary |
| Muehling, 2009 | Germany | Single, 96 | Vascular | General, neuraxial | Protocol or guidelines implementation | Preoperative, Intraoperative, Postoperative | mean: 68, range: 52-84; mean: 67, range: 40-81 | 97% | ASA II: 5, ASA III: 34, ASA IV: 3; ASA II: 5, ASA III: 32, ASA IV: 0 | Mortality, in-hospital, 30 days, primary |
| Mullis-Jansson, 1999 | USA | Single, 170 | Cardiac | General, sedation, other: Vecuronium bromide | Pharmacotherapy | Intraoperative | mean: 66.0, SD: 10.8; mean: 62.1, SD: 12.5 | 82% | ASA II: 5, ASA III: 34, ASA IV: 3; ASA II: 5, ASA III: 32, ASA IV: 0 | Death, NR, NR |
| Murkin, 2007 | NR | Single, 200 | Cardiac | General | Device | Intraoperative | mean: 61.8, SD: 10.3; mean: 61.8, SD: 9.3 | 88% | NR | Death, 30 days, primary |
| Namikawa, 2013 | Japan | Single, 26 | Oncological | General | Pharmacotherapy | Postoperative | mean: 78, range: 70-92; mean: 78, range: 74-83 | 50% | NR | Mortality, in-hospital, primary |
| Nathan, 1991 | UK | Single, 197 | General | General | Device | Postoperative | mean: 70, SD: 7; mean: 68, SD: 6 | 63% | NR | Mortality, in-hospital, NR |
| Nathan, 2003 | Canada | Single, 144 | Cardiac | General | Temperature | Intraoperative | NR | NR | NR | Mortality, in-hospital, secondary |
| Nejad, 2011 | Iran | Single, 150 | Cardiac | General | Pharmacotherapy | Intraoperative | mean: 54.2, SD: 9.7; mean: 53.6, SD: 9.1; mean: 54.6, SD: 10.4; | 77% | NR | Mortality, in-hospital, secondary |
| Neškoviü, 2013 | Serbia | Single, 82 | Cardiac | General, neuraxial | Anesthetic technique | Intraoperative | mean: 51.9, SD: 7.6; mean: 56.9, SD: 8.5; mean: 55.4, SD: 7.9; mean: 55.0, SD: 7.8 | 83% | NR | Mortality, NR, secondary |
| Newman, 2012 | Multiple | Multi-site, 300, 2986 | Cardiac | General | Pharmacotherapy | Preoperative, Intraoperative, Postoperative | mean: 66.7, SD: 8.7; mean: 66.2, SD: 8.5 | 70% | NR | All-cause mortality, in-hospital, 28 days, primary |
| Ng, 2008 | Hong Kong | Single, 50 | Cardiac | General | Ventilation related | Intraoperative | mean: 59, SD: 8; mean: 63, SD: 11 | 64% | NR | Death, NR, NR |
| Nguyen, 2010 | USA | Multi-site, 30, 1000 | Neurological | General | Temperature | Intraoperative | mean: 51, SD: 13; mean: 52, SD: 12 | NR | NR | Mortality, in-hospital, 90 days, NR |
| Niederhauser, 1997 | Switzerland | Single, 53 | Cardiac | General | Pharmacotherapy | Intraoperative, Postoperative | NR | 79% | NR | Mortality, in-hospital, 60 days, primary |
| Nomura, 2007 | Japan | Single, 64 | General | General | Nutritional | Preoperative, Postoperative | mean: 69, range: 50-88; mean: 66, range: 30-83 | 61% | NR | Mortality, in-hospital, secondary |
| Norgren, 2004 | Sweden | Multi-site, 20, 817 | Vascular | General | Pharmacotherapy | Intraoperative | NR | 62% | NR | Mortality, in-hospital, 30 days, secondary |
| Norman, 2009 | USA | Single, 20 | Thoracic | General | Pharmacotherapy | Intraoperative | mean: 62, SD: 7.6; mean: 63.5, SD: 6.2 | 85% | NR | Mortality in patients evaluable for disease progression, 3 years, secondary  Survival, 3 years, secondary |
| Norris, 2001 | USA | Single, 168 | Vascular | General, neuraxial | Pharmacotherapy | Intraoperative, Postoperative | mean: 70, SD: 9.5; mean: 67, SD: 10; mean: 68, SD: 9.9; mean: 68, SD: 8.4 | 68% | NR | Death during hospital stay, in-hospital, secondary  Cardiac death, 1 year, secondary  Mortality, 1 year, secondary |
| Nussmeier, 2005 | Multiple | Multi-site, 175, 1671 | Cardiac | General | Pharmacotherapy | Postoperative | mean: 62.1, SD: 8.6; mean: 61.6, SD: 9.1; mean: 62.0, SD: 9.1; | 86% | NR | Death, NR, secondary |
| Ochroch, 2006 | USA | Single, 2531 | Cardiac | General | Monitoring | Postoperative | mean: 59.9, SD: 15.3; mean: 61.8, SD: 13.3 | 62% | NR | Mortality, in-hospital, secondary |
| Oda, 2004 | Japan | Single, 40 | Thoracic | General | Pharmacotherapy | Intraoperative, Postoperative | mean: 62, SD: 8; mean: 59, SD: 9 | 90% | Levels aggregated: Median ASA score: 3; Levels aggregated: Median ASA score: 3 | Death, NR, secondary |
| Okabayashi, 2009 | Japan | Single, 30 | General | General | Glucose | Postoperative | mean: 63.2, SD: 7.5; mean: 61.9, SD: 13.6 | 60% | NR | Mortality, in-hospital, 30 days, secondary |
| Onorati, 2006 | Italy | Single, 50 | Cardiac | General | Device | Intraoperative | mean: 69.6, SD: 9.5; mean: 72.2, SD: 5.7 | 76% | NR | In-hospital mortality, in-hospital, 30 days, primary |
| Ovrum, 2009 | Norway | Single, 399 | Cardiac | General | Pharmacotherapy | Intraoperative, Postoperative | mean: 66, range: 32-86; mean: 66, range: 35-87 | NR | NR | 30d mortality, in-hospital, 30 days, secondary |
| Papadopoulos, 2010 | Greece | Single, 50 | Cardiac | General | Pharmacotherapy | Intraoperative, Postoperative | mean: 62, SD: 15, range: 32-81; mean: 66, SD: 13, range: 32-81 | 82% | NR | Death, in-hospital, 2 days, 88 hours, 4 days, secondary |
| Pargger, 1998 | Switzerland | Single, 55 | Vascular | General | Protocol or guidelines implementation | Postoperative | mean: 67, SD: 9; mean: 64, SD: 10 | NR | NR | Hospital mortality, in-hospital, secondary |
| Park, 2001 | USA | Multi-site, 15, 984 | Colorectal, general or vascular | General, neuraxial | Anesthetic technique | Intraoperative, Postoperative | mean: 67.0, SD: 8.8; mean: 66.5, SD: 8.9 | NR | Levels aggregated: Mean ASA 3 +/- 0.6; Levels aggregated: Mean ASA 3, +/- 0.5 | 30-day mortality, in-hospital, 30 days, primary |
| Parke, 2013 | New Zealand | Single, 340 | Cardiac | General | Ventilation related | Postoperative | mean: 66, range: 21-87; mean: 65, range: 19-88 | 76% | ASA III: 460, ASA IV: 47; ASA III: 471, ASA IV: 42 | Mortality, 28 days, secondary |
| Parker, 2010 | UK | Single, 243 | Orthopedic | NR | Pharmacotherapy | Postoperative | Mean: 83, Range: 61-104; Mean: 81, Range: 60-96 | 23% | NR | Mortality at 30 days, 90 days, 120 days, secondary |
| Parker, 2004 | UK | Single, 396 | Orthopedic | General, neuraxial | Pharmacotherapy | Preoperative | mean: 50.8, range: 46-98; mean: 80.9, range: 43-100 | 20% | NR | 30d mortality, 30 days, primary  Mortality, 60 days, primary  Mortality, 120 days, primary  Mortality, 1 year, primary |
| Pattanshetty, 2015 | India | Single, 173 | NR | NR | Physio | Postoperative | mean: 49.7, SD: 16.21; mean: 49.4, SD: 16.13 | 76% | Levels aggregated: I or II - 61; Levels aggregated: I or II - 48 | Mortality, NR, NR |
| Pearse, 2005 | UK | Single, 122 | General, urology, vascular | General | Pharmacotherapy | Postoperative | mean: 68, SD: 11.6; mean: 66, SD: 11.4 | 66% | NR | 28-day mortality, 28 days, secondary  Mortality, 60 days, secondary |
| Pearse, 2014 | UK | Multi-site, 17, 734 | Major gastrointestinal surgery | General | IV fluids | Intraoperative, Postoperative | Mean: 71.3 (8.4); mean: 72.2 (8.6) | 98% | ASA 1: 5.7, 6.6; ASA 2: 54.5, 48.1; ASA 3: 39, 42.8; ASA 4: 0.8, 2.5 | Composite 30-day moderate/major complications and mortality, primary  All-cause mortality at 30 and 180 days, secondary |
| Pestaña, 2014 | Multiple | Multi-site, 6, 133 | Colorectal | General, neuraxial | Monitoring | Intraoperative, Postoperative | mean: 74, range: 64-79; mean: 73.5, range: 63.5-80 | 60% | Levels aggregated: 72% ASA 3 and above; Levels aggregated: 81% ASA 3 and above | Hospital mortality, in-hospital, secondary |
| Pexe-Machado, 2013 | Brazil | Single, 22 | Colorectal | General, neuraxial | Nutritional | Preoperative | mean: 48, SD: 12; mean: 49, SD: 10 | 36% | ASA I: 2, ASA II: 34, ASA III: 34, ASA IV: 0; ASA I: 2, ASA II: 31, ASA III: 37, ASA IV: 2 | Overall mortality, NR, secondary |
| Pinheiro de Almeida, 2015 | Brazil | Single, 198 | Oncological | General, neuraxial | Transfusion related | Postoperative | mean: 64, SD: 14; mean: 64, SD: 12 | 56% | ASA I: 5(41.7), ASA II: 6(50), ASA III: 1(8.3); ASA I: 1(10), ASA II: 7(70), ASA III: 2(20) | Primary composite outcome, 30 days, primary  Mortality from all causes, 30 days, secondary  Mortality, 60 days, secondary |
| POBBLE Trial Investigators, 2005 | UK | Multi-site, 4, 97 | Vascular | General | Pharmacotherapy | Preoperative, Postoperative | mean: 74, range: 66-76; mean: 73, SD: 61-79 | 77% | ASA II: 57, ASA III: 30, ASA IV: 9, ASA V: 1; ASA II: 67, ASA III: 25, ASA IV: 8, ASA V: 1 | Fatal and nonfatal cardiovascular events, 30 days, primary  Mortality from any cause, 30 days, secondary  Survival, 2 years, secondary |
| Poldermans, 1999* | Multiple | Multi-site, 7, 112 | Vascular | NR | Pharmacotherapy | Preoperative, Intraoperative, Postoperative | mean: 67, range: 61-75; mean: 68, range: 60-73 | 79% | NR | Perioperative death, in-hospital, primary |
| Poldermans, 2006* | Multiple | Multi-site, 5, 1467 | Vascular | General | Testing | Preoperative | mean: 68.0, range: 60.9-73.5; mean: 67.3, range: 60.9-73.9 | 73% | NR | Cardiac death (as part of composite score), 30 days, primary |
| Potočnik, 2014 | Slovenia | Single, 36 | Thoracic | General | Pharmacotherapy | Intraoperative | mean: 60.9, SD: 9.4; mean: 52.7, SD: 14.6 | 50% | NR | Death, NR, secondary |
| Prowle, 2012 | Australia | Single, 85 | Cardiac | General | Pharmacotherapy | Preoperative, Postoperative | mean: 67.3, SD: 10.8; mean: 69, SD: 11.1 | 82% | NR | Hospital mortality, in-hospital, NR |
| Pupelis, 2000 | Latvia | Single, 29 | General | NR | Nutritional | Postoperative | mean: 50, SD: 10; mean: 53, SD: 11 | NR | NR | Death, in-hospital, secondary |
| Qiu, 2009 | China | Single, 221 | Cardiac | General | Device | Intraoperative | age over 65 n, %: 78(67.8); 80 (75.5) | 33% | NR | Mortality, in-hospital, NR |
| Rabie, 2006 | Saudi Arabia | Single, 20 | General | General | Pharmacotherapy | Intraoperative | mean: 26.8, SD: 5.3; mean: 24.6, SD: 4.5, range: 18-35 | 85% | NR | Preoperative Mortality, NR, secondary |
| Racette, 1987 | USA | Single, 56 | Colorectal | General | Device | Postoperative | mean: 63; mean: 69 | 36% | ASA I: 10; ASA I: 10 | Death, in-hospital, secondary |
| Rao, 2002 | Canada | Multi-site, 2, 1127 | Cardiac | General | Pharmacotherapy | Intraoperative | mean: 63, SD: 10; mean: 64, SD: 11 | 74% | NR | Operative mortality, in-hospital, secondary |
| Rasmussen, 2006 | NR | Multi-site, NR, 36 | Orthopedic | General, neuraxial | Pharmacotherapy | Intraoperative | mean: 70, SD: 6; mean: 71, SD: 8 | 36% | NR | Deaths, 90 days, secondary |
| Rayes, 2007 | Germany | Single, 80 | General | NR | Nutritional | Preoperative, Postoperative | mean: 59, SD: 13; mean: 58, SD: 12 | 56% | NR | Perioperative mortality, in-hospital, NR |
| Reggiori, 1996 | Uganda | Single, 850 | General | NR | Pharmacotherapy | Intraoperative, Postoperative | NR | NR | ASA I: 3, ASA II: 27, ASA III: 10, ASA IV: 0; ASA I: 3, ASA II: 21, ASA III: 16, ASA IV: 0 | Mortality, NR, NR |
| Reis, 2002 | Portugal | Single, 264 | Cardiac | General | Protocol or guidelines implementation | Postoperative | mean: 62, SD: 9; mean: 63, SD: 10 | NR | NR | 30-day mortality, 30 days, NR |
| Reyad, 2013 | Egypt | Single, 60 | General | General | Pharmacotherapy | Intraoperative | mean: 40.44, SD: 8.11; mean: 42.90, SD: 6.78; mean: 41.12, SD: 7.34; | NR | NR | Death, in-hospital, secondary |
| Reyes, 1997 | Spain | Single, 404 | Cardiac | General | Ventilation related | Postoperative | mean: 61.0, SD: 11.3; mean: 61.0, SD: 10.7 | 68% | ASA I: 10%, ASA II: 35%, ASA III: 35%; ASA I: 10%, ASA II: 30%, ASA III: 60%; ASA I: 10%, ASA II: 35%, ASA III: 55% | Overall mortality, 30 days, secondary |
| Rideout, 2011 | UK | Single, 110 | Cardiac | General | Physio | Preoperative | NR | NR | NR | Survival/mortality, 12 years, primary |
| Robinson, 2000 | Multiple | Multi-site, 9, 79 | Vascular | NR | Pharmacotherapy | Intraoperative | mean: 73, range: 52/86; mean: 74, range: 56-88 | 84% | NR | Death, 30 days, NR |
| Rodrıguez, 2014 | Spain | Single, 121 | General | General, neuraxial | Pharmacotherapy | Intraoperative | mean: 62, SD: 20; mean: 60, SD: 21; mean: 65, SD: 12; | 48% | NR | Mortality, 30 days, secondary |
| Rujirojindakul, 2014 | Thailand | Single, 199 | Cardiac | General | Pharmacotherapy | Intraoperative, Postoperative | mean: 54; mean: 54 | 56% | ASA I: 2, ASA II: 29, ASA III: 5; ASA I: 1, ASA II: 31, ASA III: 4; ASA I: 0, ASA II: 28, ASA III: 6 | Mortality, in-hospital, secondary |
| Ryan, 1986 | Australia | Multi-site, 7, 261 | Colorectal | General | Pharmacotherapy | Preoperative, Intraoperative | mean: 65; mean: 66 | 49% | ASA III: 87, ASA IV: 10, ASA V: 3; ASA III: 86, ASA IV: 12, ASA V: 1 | Overall 30-day mortality, 30 days, secondary |
| Sajja, 2006 | India | Single, 116 | Cardiac | General | Device | Intraoperative | mean: 60.5, SD: 7.87; mean: 60, SD: 8.43 | 89% | NR | Mortality, in-hospital, NR |
| Sandham, 2003 | Canada | Multi-site, 19, 1994 | Cardiac | General | Device | Preoperative, Intraoperative, Postoperative | mean: 72.6, SD: 6.89; mean: 72.3, SD: 6.97 | 71% | NR | In hospital mortality, in-hospital, primary  Mortality, 6 months, secondary  Mortality, 1 year, secondary |
| Sandstrom, 1993 | Sweden | Single, 300 | Major surgery | General | Nutritional | Postoperative | NR | 63% | ASA III: 871, ASA IV: 126; ASA III: 871, ASA IV: 126 | Mortality, in-hospital, secondary |
| Sandven, 2002 | Norway | Multi-site, 26, 109 | General | General | Pharmacotherapy | Intraoperative | mean: 60, range: 15-87; mean: 68, range: 13-86 | 48% | NR | Mortality, 3 months, primary |
| Sarac, 1998 | USA | Multi-site, 2, 56 | Vascular | General | Pharmacotherapy | Preoperative, Postoperative, after discharge from hospital | mean: 66.2; mean: 69.4 | 79% | NR | Perioperative mortality rate, in-hospital, 30 days, primary  Cumulative 5-year survival, 5 years, NR |
| Saran, 2011 | UK | Single, 192 | Thoracic | General | Pharmacotherapy | Intraoperative, Postoperative | mean: 64.0, range: 56-71; mean: 65.5, range: 58-70 | 57% | NR | Inpatient mortality, in-hospital, secondary  Mortality, 30 days, secondary |
| Schaefer, 2004 | Germany | Multi-site, 5, 153 | Thoracic | General | Pharmacotherapy | Preoperative, Postoperative | mean: 60.2, range: 38-74; mean: 58.7, range: 29-76 | 82% | ASA I: 3, ASA II: 66, ASA III: 27; ASA I: 4, ASA II: 53, ASA III: 39 | In hospital mortality, in-hospital, secondary  Mortality, 30 days, secondary |
| Schubert, 2003 | USA | Multi-site, 19, 173 | General, orthopedic or vascular | General, neuraxial | Transfusion related | Intraoperative, Postoperative | mean: 68, SD: 13; mean: 65, SD: 16 | NR | NR | Mortality, NR, secondary |
| Segers, 2006 | Netherlands | Single, 954 | Cardiac | General | Pharmacotherapy | Preoperative, Postoperative | mean: 66.4, SD: 9.9; mean: 65.3, SD: 10.4 | 73% | ASA I: 0, ASA II: 19.1, ASA III: 80.9; ASA I: 5.4, ASA II: 29.3, ASA III: 65.2 | Mortality, in-hospital, secondary |
| Senagore, 2009 | USA | Single, 64 | Colorectal | NR | IV Fluids | Intraoperative | NR | NR | NR | Operative mortality, in-hospital, NR |
| Serclova , 2009 | Czech Republic | Single, 103 | General | General, neuraxial | Protocol or guidelines implementation | Preoperative, Intraoperative, Postoperative | mean: 37.6, SD: 12.5; mean: 35.1, SD: 11 | 50% | NR | Deaths, NR, secondary |
| Serrano-Trenas, 2011 | Spain | Single, 179 | Orthopedic | General, neuraxial | Transfusion related | Preoperative, Postoperative | mean: 82.53, SD: 6.37; mean: 53.46, SD: 7.11 | NR | NR | Mortality, in-hospital, 30 days, secondary |
| Sethi, 1990 | USA | Multi-site, 12, 772 | Cardiac | General | Pharmacotherapy | Preoperative, Postoperative | mean: 58, SD: 7; mean: 59, SD: 8 | 26% | ASA II: 24, ASA III: 70; ASA II: 22, ASA III: 73 | Mortality, in-hospital, 30 days, NR |
| Sezai, 2010 | Japan | Single, 133 | Cardiac | General | Pharmacotherapy | Intraoperative, Postoperative | mean: 65.9, SD: 8.9; mean: 65.9, SD: 10.4 | 86% | NR | 30d death, 30 days, primary  Death, 180 days, primary  Late postoperative death, NR, primary |
| Sezai, 2011 | Japan | Single, 140 | Cardiac | General | Pharmacotherapy | Intraoperative, Postoperative | mean: 66.3, SD: 0.6; mean: 65.6, SD: 0.6 | 79% | NR | Intraoperative mortality, NR, secondary |
| Sezai, 2013 | Japan | Single, 367 | Cardiac | NR | Pharmacotherapy | Intraoperative | mean: 70.8, SD: 8.8; mean: 70.1, SD: 8.4 | 73% | NR | Death, 2 year, secondary  Cardiac death, 2 years, secondary |
| Sezai, 2009 | Japan | Multi-site, 7, 504 | Cardiac | General | Pharmacotherapy | Intraoperative, Postoperative | mean: 66.7, SD: 8.9; mean: 68.5, SD: 4.7 | 25% | NR | Post-operative death, in-hospital, NR |
| Shackford, 1961 | USA | Single, 35 | Vascular | General | Protocol or guidelines implementation | Postoperative | mean: 59, SD: 2; mean: 62, SD: 2 | NR | NR | Mortality, in-hospital, NR |
| Shah, 2012 | Nepal | Single, 328 | General | General | Pharmacotherapy | Preoperative | mean: 41.6, range: 10-76; mean: 40.3, range: 13-76 | 21% | Levels aggregated: Mean ASA 2.8 +/- 0.2; Levels aggregated: Mean ASA 2.7 +/- 0.1 | Mortality, NR, secondary |
| Shangraw, 2008 | USA | Single, 250 | General | General | Pharmacotherapy | Intraoperative | mean: 47, SD: 9; mean: 49, SD: 9 | 72% | NR | Mortally, 7 days, 30 days, secondary |
| Shukla, 2008 | India | Single, 99 | Oncological | General | Pharmacotherapy | Preoperative, Postoperative | NR | NR | NR | Postoperative deaths, NR, secondary |
| Sinclair, 1997 | UK | Single, 40 | Orthopedic | General, regional | IV Fluids | Intraoperative | NR | NR | NR | Mortality, in-hospital, secondary |
| Singh, 1998 | India | Single, 43 | General | NR | Nutritional | Postoperative | mean: 40.8, SD: 2.3; mean: 38.9, SD: 2.1 | NR | Levels aggregated: 2 (2-3); Levels aggregated: 2 (2-3) | Mortality, in-hospital, NR |
| Sirivella, 1998 | USA | Single, 298 | Cardiac | General | Pharmacotherapy | Postoperative | mean: 69, SD: 4.5; mean: 71, SD: 4 | 80% | NR | Hospital mortality, 30 days, NR |
| Sirlak, 2004 | Turkey | Single, 80 | Cardiac | General | Pharmacotherapy | Preoperative, Intraoperative, Postoperative | mean: 66, SD: 2.8; mean: 65, SD: 3.2 | NR | NR | Mortality, in-hospital, secondary |
| Skånberg, 2009 | Sweden | Multi-site, 7, 640 | Oncological | General | Transfusion related | Preoperative, Intraoperative, Postoperative | mean: 73, range: 36-91; mean: 75, range: 37-90; mean: 70, range: 34-90; | NR | NR | Number of death or survival rate, 10 years, primary  Median cumulative survival time, 10 years, primary |
| Skhirtladze, 2014 | Austria | Single, 236 | Cardiac | General | IV Fluids | Intraoperative | NR | 70% | NR | Mortality, 90 days, NR |
| Smith, 2010 | Multiple | Multi-site, 249, 4117 | Cardiac | General | Pharmacotherapy | Intraoperative, Postoperative | mean: 66.2; mean: 66.2 | NR | NR | Death, 30 days, 90 days, 180 days, primary |
| Soares, 2009 | Brazil | Single, 40 | Cardiac | General | IV Fluids | Intraoperative | mean: 67, SD: 11; mean: 66, SD: 10 | 70% | NR | Mortality, NR, secondary |
| Spahn, 2002 | Multiple | Multi-site, 34, 468 | Non-cardiac | NR | Transfusion related | Intraoperative | mean: 59, SD: 13; mean: 59, SD: 11 | NR | NR | Mortality, in-hospital, 90 days, secondary |
| Spotnitz, 2013 | USA | Single, 27 | Cardiac | General | Device | Intraoperative, Postoperative | NR | NR | ASA I: 22, ASA II: 63, ASA III: 15; ASA I: 15, ASA II: 72, ASA III: 13 | 30-day mortality, 30 days, secondary |
| Squadrone, 2005 | Italy | Multi-site, 15, 209 | General | General | Ventilation related | Postoperative | mean: 65, SD: 10; mean: 66, SD: 9 | 65% | NR | Death, in-hospital, secondary |
| Stone, 2003 | UK | Single, 100 | Colorectal, general, or oncological urology | General, neuraxial | Pharmacotherapy | Intraoperative, Postoperative | mean: 69, range: 63-77; mean: 70, range: 63-77 | 71% | NR | Mortality, in-hospital, primary |
| Subramaniam, 2009 | USA | Single, 236 | Orthopedic or vascular | General | Pharmacotherapy | Intraoperative, Postoperative | mean: 67, SD: 10; mean: 71, SD: 11 | 56% | NR | Death, in-hospital, NR |
| Suezawa, 2013 | Japan | Single, 30 | Cardiac | General | Pharmacotherapy | Intraoperative | mean: 71, SD: 7; mean: 66, SD: 11 | 77% | ASA II: 4 (3.3), ASA III: 103 (84.4), ASA IV: 15 (12.3); ASA II: 10 (8.5), ASA III: 94 (83), ASA IV: 10 (8.5) | Deaths, in-hospital, secondary |
| Sufit, 2012 | USA | Multi-site, 3, 10 | Cardiac | General | Pharmacotherapy | Preoperative | mean: 56.67, SD: 8.17; mean: 65.25, SD: 8.96 | 80% | NR | Day 28 mortality, 28 days, primary |
| Takala, 2000 | Multiple | Multi-site, 13, 412 | General | General | Pharmacotherapy | Preoperative, Intraoperative, Postoperative | mean: 62.5, SD: 13.5; mean: 61.2, SD: 14.5; mean: 63.8, SD: 12.2; | 63% | NR | Overall mortality, NR, secondary  Death, NR, secondary |
| Tamayo, 2008 | Spain | Single, 44 | Cardiac | General | IV Fluids | Intraoperative | mean: 66.50, SD: 7.5; mean: 67.8, SD: 8.1 | 82% | NR | Death, in-hospital, 30 days, secondary |
| Thielmann, 2005 | Germany | Single, 57 | Cardiac | General | Pharmacotherapy | Intraoperative, Postoperative | mean: 65, SD: 9; mean: 64, SD: 10 | 77% | NR | All-cause mortality, 30 days, 1 year, secondary |
| Thielmann, 2013 | Germany | Single, 329 | Cardiac | General | Device | Intraoperative | mean: 69.1, SD: 9.2; mean: 68.2, SD: 10.3 | 82% | NR | Cardiac death, 30 days, secondary  Cardiac death, 1 year, secondary  Combined secondary outcomes at end of follow-up, >4years, secondary  Mortality, 2 year, secondary |
| Thomas, 2001 | UK | Single, 219 | Orthopedic | General, regional, neuraxial, other: Combinations of the above also used, depicted in Table 2. | Transfusion related | Intraoperative, Postoperative | mean: Females 70.2, males 69.7, range: Females 40-87, males 48-88; mean: Females 70.5, males 67.4, range: Females 32-95, males 38-85 | 45% | NR | Mortality, 90 days, secondary |
| Todd, 2005 | Multiple | Multi-site, 30, 1000 | Neuro | General | Temperature | Intraoperative | mean: 51, SD: 13; mean: 52, SD: 12 | 35% | Levels aggregated: Aggregated by gender, reported as mean and range. Allogenic females 2.1 (1-3), allogenic males 1.9 (1-3); Levels aggregated: Aggregated by gender, reported as mean and range. Allogenic females 2.1 (1-3), allogenic males 2.0 (1-3) | In-hospital deaths overall, in-hospital, secondary |
| Treschan, 2012 | Multiple | Single, 101 | General | General, neuraxial | Ventilation related | Intraoperative | mean: 68, SD: 9, range: 51-86; mean: 68, SD: 8, range: 52-87 | 74% | NR | Death due to sepsis, in-hospital, secondary  Death due to bleeding, in-hospital, secondary  30d mortality, 30 days, secondary |
| Turner, 2007 | UK | Single, 18 | Vascular | General | Pharmacotherapy | Preoperative | mean: 69.1, SD: 5.4; mean: 71.9, SD: 6.0 | NR | ASA II: 14, ASA III: 35, ASA IV: 2; ASA II: 15, ASA III: 34, ASA IV: 1 | Death, NR, secondary |
| Turner, 2014 | USA | Multi-site, 2, 120 | Cardiac | General | Pharmacotherapy | Intraoperative, Postoperative | mean: 69.7, SD: 13.5; mean: 70.2, SD: 12.6 | 64% | NR | Death, 30 days, secondary |
| Turpie, 2007 | USA | Multi-site, 50, 842 | General | General, neuraxial | Pharmacotherapy | Postoperative | range: 40-95; range: 40-93 | 75% | NR | Death in ICU, in-hospital, primary |
| Van de Watering, 1998 | Netherlands | Single, 914 | Cardiac | General | Transfusion | Preoperative | Mean: 64.4, SD: 9.5; mean: 62.9, SD:9.8; mean: 63.3, SD:9.1 | 71% | NR | Morality, until day 60, secondary |
| Van Den Berghe, 2001 | Belgium | Single, 1548 | NR | NR | Glucose | Postoperative, other: ICU population mixed surgical and medical | mean: 62.2, SD: 13.9; mean: 63.4, SD: 13.6 | 71% | NR | Mortality, in-hospital, secondary  Mortality, 30 days, secondary |
| van Mastrigt, 2005 | Netherlands | Single, 597 | Cardiac | General | Monitoring | Postoperative | mean: 62.6, SD: 9.42%; mean: 62.7, SD: 9.30% | 80% | NR | Mortality, 30 days, secondary |
| Veneman, 2004 | Netherlands | Single, 61 | NR | NR | Pharmacotherapy | Postoperative | mean: 67; mean: 72; mean: 68; mean: 71 | 59% | NR | Mortality, in-hospital, NR |
| Venn, 2001 | UK | Single, 20 | Major abdominal surgery | General, regional | Pharmacotherapy | Postoperative | mean: 84.5, SD: 9.3, range: 65-102; mean: 85, SD: 6.2, range: 74-98; mean: 82, SD: 8.7, range: 65-97; | 80% | NR | Mortality, NR, secondary |
| Venn, 2002 | UK | Single, 90 | Orthopedic | NR | IV Fluids | Intraoperative | mean: 67, range: 64-74; mean: 65, range: 60-77 | NR | Levels aggregated: 3 (3-4); Levels aggregated: 3 (3-4); Levels aggregated: 3 (2.5-3) | Death, in-hospital, secondary |
| Vichinsky, 1995 | USA | Multi-site, 36, 604 | Multiple | General | Transfusion related | Preoperative | range: 0-9 yo 40; 10-19 yo-36; >20 yo 24; range: 0-9 yo 40; 10-19 yo-35; >20 yo 25 | 51% | NR | In-hospital mortality, in-hospital, secondary |
| Vlug, 2011 | Netherlands | Multi-site, 9, 400 | Oncological | NR | Protocol or guidelines implementation | Preoperative, Intraoperative, Postoperative | mean: 66, SD: 7.1; mean: 68, SD: 8.8; mean: 66, SD: 10.3; mean: 66, SD: 8.6 | 58% | ASA II: 48, ASA III: 51, ASA IV: 1; ASA II: 47, ASA III: 51, ASA IV: 2 | Death, NR, secondary |
| Wadhwa, 2014 | Multiple | Multi-site, 3, 400 | Bariatric | General | FiO2 | Postoperative | mean: 43, SD: 12; mean: 45, SD: 12 | 21% | Levels aggregated: 77% (ASA I-II); Levels aggregated: 80% (ASA I-II); Levels aggregated: 81% (ASA I-II) | 30d mortality, 30 days, NR |
| Wallace, 2004 | USA | Single, 190 | Non-cardiac | General | Pharmacotherapy | Preoperative, Intraoperative, Postoperative | mean: 69.2, SD: 8.7; mean: 68.0, SD: 7.5 | NR | ASA I: 13 (7), ASA II: 67 (34), ASA III: 11(56), ASA IV: 6 (3); ASA I: 9 (4), ASA II: 75 (37), ASA III: 110 (55), ASA IV: 8 (4) | Mortality, 2 years, NR  In hospital mortality, in-hospital, secondary |
| Wang, 2012 | China | Single, 7 | General | General | Protocol or guidelines implementation | Preoperative, Intraoperative, Postoperative | mean: 55.4, SD: 16.8; mean: 56.1, SD: 14.6; mean: 57.2, SD: 18.1; mean: 55.7, SD: 17.3 | 57% | NR | 28-day mortality, 30 days, NR |
| Wang, 2012 | China | Multi-site, 2, 457 | Non-cardiac | NR | Pharmacotherapy | Postoperative | mean: 74.4, SD: 7; mean: 74, SD: 5.8 | 63% | ASA I: 16, ASA II: 20, ASA III: 6; ASA I: 14, ASA II: 22, ASA III: 4; ASA I: 17, ASA II: 18, ASA III: 6 | Mortality, in-hospital, 90 days, secondary |
| Webb, 1997 | UK | Single, 96 | Cardiac | General | Pharmacotherapy | Preoperative, Postoperative | mean: 61, range: 39-77; mean: 62, range: 44-78 | 90% | ASA II: 135, ASA III: 88, ASA IV: 5; ASA II: 145, ASA III: 81, ASA IV: 3 | Death at 28 days, 28 days, secondary |
| Weis, 2009 | Germany | Single, 36 | Cardiac | General | Pharmacotherapy | Intraoperative, Postoperative | mean: 69, range: 63-72; mean: 67, range: 61-78 | 58% | NR | 45-day mortality, 45 days, secondary |
| Weltert, 2013 | Italy | Single, 1049 | Cardiac | NR | Transfusion related | Intraoperative, Postoperative | mean: 66.4, SD: 9.8; mean: 68.3, SD: 9.6 | 74% | NR | Post-operative mortality, 30 days, NR |
| White, 1980 | South Africa | Single, 56 | Orthopedic | General, regional, neuraxial | Anesthetic technique | Intraoperative | mean: 80, SD: 9.1; mean: 78, SD: 7.8; mean: 78, SD: 7.3; | 14% | NR | Mortality, 30 days, secondary |
| Wichmann, 2007 | Germany | Multi-site, 4, 256 | General | General | Nutritional | Postoperative | mean: 59.1, SD: 11.5; mean: 59.6, SD: 11.6 | 56% | ASA I: 1, ASA II: 10, ASA III: 7, ASA IV: 2; ASA I: 2, ASA II: 6, ASA III: 10, ASA IV: 2; ASA I: 1, ASA II: 6, ASA III: 9, ASA IV: 0 | All cause death, NR, secondary |
| Wijeysundera, 2014 | Canada | Multi-site, 3, 168 | Non-cardiac | General, regional, neuraxial | Pharmacotherapy | Preoperative, Intraoperative, Postoperative | mean: 72, SD: 8; mean: 70, SD: 8 | NR | NR | Hospital mortality, in-hospital, primary |
| Wilson, 1999 | UK | Single, 138 | General, urology, vascular | General | Pharmacotherapy | Preoperative, Intraoperative, Postoperative | NR | NR | NR | Death, NR, secondary |
| Wojciech-Szczeklik, 2010 | Poland | Single, 24 | Cardiac | General | Pharmacotherapy | Intraoperative | mean: 59.6, SD: 6.8; mean: 55.6, SD: 6.9 | 83% | NR | Mortality, NR, NR |
| Wolowczyk, 2005 | UK | Single, 36 | Vascular | General | Transfusion related | Intraoperative, Postoperative | range: 60-87; range: 55-83 | 64% | NR | Mortality, in-hospital, secondary |
| Wu, 2006 | Taiwan | Single, 212 | Oncological | General | Pharmacotherapy | Preoperative, Intraoperative, Postoperative | mean: 62, range: 22-88; mean: 57, range: 28-84 | 74% | NR | Mortality, in-hospital, NR |
| Wu, 2006 | China | Single, 468 | Oncological | NR | Nutritional | Preoperative, Postoperative | mean: 56.5, range: 24-86; mean: 57.3, range: 21-84 | 70% | NR | 30- and 90- day mortality, 30 days, 90 days, secondary |
| Wuethrich, 2014 | Switzerland | Single, 166 | Urology | General, neuraxial | IV Fluids | Intraoperative | mean: 69, range: 42–88; mean: 68, range: 38–88 | 69% | NR | One-year mortality, 1 year, secondary |
| Yang, 2006 | Canada | Multi-site, 3, 497 | Vascular | General | Pharmacotherapy | Preoperative, Intraoperative, Postoperative | mean: 65.9, SD: 10; mean: 66.4, SD: 10 | 76% | NR | Cardiac death, 30 days, primary  Non-cardiac death, 30 days, NR |
| Zakhaleva, 2012 | USA | Single, 74 | Colorectal | General | IV Fluids | Intraoperative | mean: 57, range: 27-79; mean: 57, range: 22-80 | 54% | ASA I: 0, ASA II: 91, ASA III: 159; ASA I: 1, ASA II: 92, ASA III: 152 | 30d mortality, in-hospital, 30 days, secondary |
| Zheng, 2010 | China | Single, 100 | Cardiac | General | Glucose control | Intraoperative, Postoperative | mean: 44.0, SD: 11.5; mean: 43.3, SD: 11.7 | 47% | ASA II: 7, ASA III: 32; ASA II: 7, ASA III: 26 | Hospital death, in-hospital, secondary |
| Zhi-ying, 2010 | China | Single, 40 | General | General | Pharmacotherapy | Intraoperative, Postoperative | mean: 42.2, SD: 13.7; mean: 47.4, SD: 8.5 | 83% | NR | Mortality, 90 days, NR |
| Zhu, 2013 | China | Single, 95 | Cardiac | NR | Ventilation | Postoperative | Mean: 62, SD: 10.3; mean: 61, SD: 12.2 | 62% | APACHE II Score: 17.2 +/-3.6; 17.6+/-3.5 | In-hospital mortality, primary |
| *This study was part of an investigation of academic integrity. The investigating committee was unable to confirm or deny any doubts surrounding the conduct of the study and it thus not retracted from the journal where it was published. We therefore did not exclude the study from our scoping review. The full report can be found here: https://www.erasmusmc.nl/1172194/2014/4771610 | | | | | | | | | | |
