# Supplementary material for: Anesthesia interventions that alter perioperative mortality: a scoping review
Source: Syst Rev. 2018 Nov 30;7:218. doi: 10.1186/s13643-018-0863-x (PMC6267894; doi:10.1186/s13643-018-0863-x)
Supplement: Supplementary file 4 — PRISMA Checklist. (PPT 2341 kb) [file 13643_2018_863_MOESM4_ESM.ppt]

## Slide 1
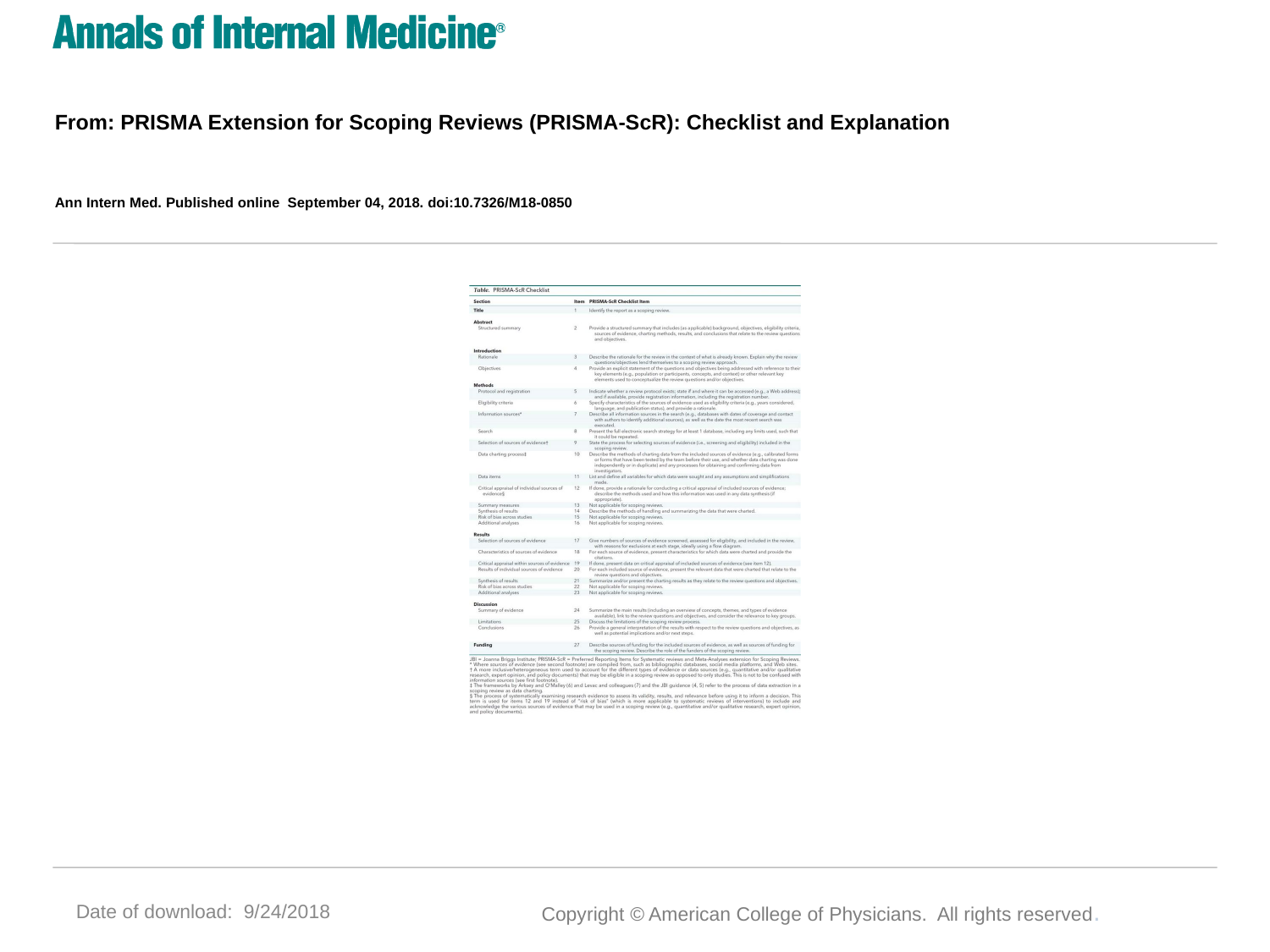

From: PRISMA Extension for Scoping Reviews (PRISMA-ScR): Checklist and Explanation
Ann Intern Med. Published online September 04, 2018. doi:10.7326/M18-0850
Date of download: 9/24/2018
Copyright © American College of Physicians. All rights reserved.
